# Inactivation of AUF1 in Myeloid Cells Protects From Allergic Airway and Tumor Infiltration and Impairs the Adenosine-Induced Polarization of Pro-Angiogenic Macrophages

**DOI:** 10.3389/fimmu.2022.752215

**Published:** 2022-02-11

**Authors:** Sofia Gargani, Niki Lourou, Christina Arapatzi, Dimitris Tzanos, Marania Saridaki, Esmeralda Dushku, Margarita Chatzimike, Nikolaos D. Sidiropoulos, Margarita Andreadou, Vasileios Ntafis, Pantelis Hatzis, Vassiliki Kostourou, Dimitris L. Kontoyiannis

**Affiliations:** ^1^ Biomedical Sciences Research Centre “Alexander Fleming”, Institute of Fundamental Biomedical Research, Vari, Greece; ^2^ Department of Genetics, Development and Molecular Biology, School of Biology, Aristotle University of Thessaloniki, Thessaloniki, Greece

**Keywords:** inflammation, innate immunity, post-transcriptional regulation, RNA-binding proteins, animal models

## Abstract

The four isoforms of the RNA-binding protein hnRNPD/AUF1 have been proposed to limit the use of inflammatory mRNAs in innate immune cells. Mice engineered to lack AUF1s in all tissues are sensitive to acute inflammatory assaults; however, they also manifest complex degenerations obscuring assessment of AUF1s’ roles in innate immune cells. Here, we restricted a debilitating AUF1 mutation to the mouse myeloid lineage and performed disease-oriented phenotypic analyses to assess the requirement of AUF1s in variable contexts of innate immune reactivity. Contrary to the whole-body mutants, the myeloid mutants of AUF1s did not show differences in their susceptibility to cytokine storms occurring during endotoxemia; neither in type-I cell-mediated reactions driving intestinal inflammation by chemical irritants. Instead, they were resistant to allergic airway inflammation and displayed reductions in inflammatory infiltrates and an altered T-helper balance. The *ex-vivo* analysis of macrophages revealed that the loss of AUF1s had a minimal effect on their proinflammatory gene expression. Moreover, AUF1s were dispensable for the classical polarization of cultured macrophages by LPS & IFNγ correlating with the unchanged response of mutant mice to systemic and intestinal inflammation. Notably, AUF1s were also dispensable for the alternative polarization of macrophages by IL4, TGFβ and IL10, known to be engaged in allergic reactions. In contrast, they were required to switch proinflammatory macrophages towards a pro-angiogenic phenotype induced by adenosine receptor signals. Congruent to this, the myeloid mutants of AUF1 displayed lower levels of vascular remodeling factors in exudates from allergen exposed lungs; were unable to support the growth and inflammatory infiltration of transplanted melanoma tumors; and failed to vascularize inert grafts unless supplemented with angiogenic factors. Mechanistically, adenosine receptor signals enhanced the association of AUF1s with the *Vegfa, Il12b, and Tnf* mRNAs to differentially regulate and facilitate the pro-angiogenic switch. Our data collectively demonstrates that AUF1s do not act as general anti-inflammatory factors in innate immune cells but have more specialized roles in regulons allowing specific innate immune cell transitions to support tissue infiltration and remodeling processes.

## Introduction

Heterogeneous nuclear ribonucleoprotein D (hnRNP D), commonly known as AU-rich element-binding factor 1 (AUF1), is an RNA-binding protein (RBP) presented in eukaryotic cells as four protein isoform members (p37, p40, p42 & p47) (1). These arise from the alternative splicing of a single pre-mRNA transcript and share two non-identical RNA-recognition motifs and a glutamine rich sequence proximal to their C-terminus. All AUF1 members can bind RNA as monomers or oligomers and localize in nuclear and cytoplasmic compartments, albeit to a variable extent (1–3).

As their name implies, AUF1 members were amongst the first RBPs identified biochemically to bind to regulatory RNA elements rich in Adenylate/Uridylate motifs (AU-Rich Elements, AREs) (4). Such elements are commonly found in the untranslated termini of mRNAs encoding immune regulators, growth signalers, and death controllers (5). Furthermore, early findings in macrophage cell lines suggested AUF1 members are post-translationally modified by immune signals to promote the degradation of pro-inflammatory mRNAs such as those encoding TNF, IL-1β, IL-3, IL-6, IL-10, GM-CSF (6–8), iNOS (9), and the NFkB regulators (10). As such, they were predicted to act in concert to other RBPs active in innate immune cells (e.g. Zfp36, Regnase-1, Roquins 1&2, TIA-1), which impede the use of inflammatory mRNAs, and whose genetic ablation in mice predisposes to acute and chronic inflammatory pathologies (4, 11–14).

Indeed, the first reports on mice deficient in AUF1s demonstrated their increased susceptibility to acute inflammatory assaults and spontaneous dermatitis upon aging (15, 16). The appearance of these phenotypes correlated to augmentations in elicited cytokine storms connecting to the increased stability of related mRNAs in immune cells. However, subsequent reports revealed these inflammatory occurrences were not due to *bona fide* aberrations in inflammation control. Instead, they seem to be triggered by tissue degenerations resulting either from their premature senescence due to involvement of AUF1 in telomere maintenance (17); or from distortion of critical developmental programs organized by AUF1 (affecting e.g. growth, myogenesis and muscle regeneration) (18–20). Notably, the fundamental roles of AUF1 in controlling aging or development were linked to the activation of key mRNAs, thus deviating from the original supposition of its action as an instructor of ARE-mediated suppression (15, 20).

Indeed, it is now clear that AUF1 members can both positively and negatively affect mRNA stability, initiation of translation, editing and even transcription (21). Comprehensive studies assessing the interactions of AUF1s revealed a relaxed stringency in their affinities for AU- to U- and GU-rich motifs (22). These studies also showed that AUF1 members do not bind only to coding but also non-coding RNAs to aid their maturation, target-loading, and function (23). The current biochemical and molecular information on AUF1s point to an indirect *modus operandi* in RNA regulation which entails changes in local RNA structures, cooperative binding with other RBPs and miRNAs or competition with other *trans*-factors (14, 21).

Still, the original findings connecting AUF1s to ARE-containing inflammatory mRNAs suggest their cell-intrinsic involvement in the regulation of innate immunity. In that context, the complex molecular activities of AUF1s could contribute to the outstanding functional heterogeneity of innate immune cells which arises due to plasticity in response to the ever-changing signals of inflamed tissues. For example, macrophages adapt to varying microenvironments and acquire a spectrum of functional phenotypes to support host defense to infection and tissue damage, different types of T-helper mediated immunity, homeostasis, and tissue vascularization and regeneration (24–27). However, the complexity of aberrations observed in whole-body AUF1 knockouts precludes judgment on the direct involvement of AUF on innate immune responses.

In this report, we identified contexts of inflammatory reactions where innate immunity was affected by the dysfunction of AUF1s. To do so, we employed a mouse system where the expression of all of AUF1 isoforms was explicitly debilitated in the myeloid lineage. Using a disease-oriented phenotypic approach, we demonstrate that AUF1s do not act as general deactivators of inflammatory responses. Instead, they are required for specific contexts of cellular immunity; and for specialized phenotypic transitions of innate immune cells, which in turn support such inflammatory contexts.

## Results

### The Susceptibility of Mice With a Germline Deletion of *hnRNPD’s* Exons 3 and 4 Differs From The Susceptibility of Mice With a Myeloid Deletion

**Figure 1:**
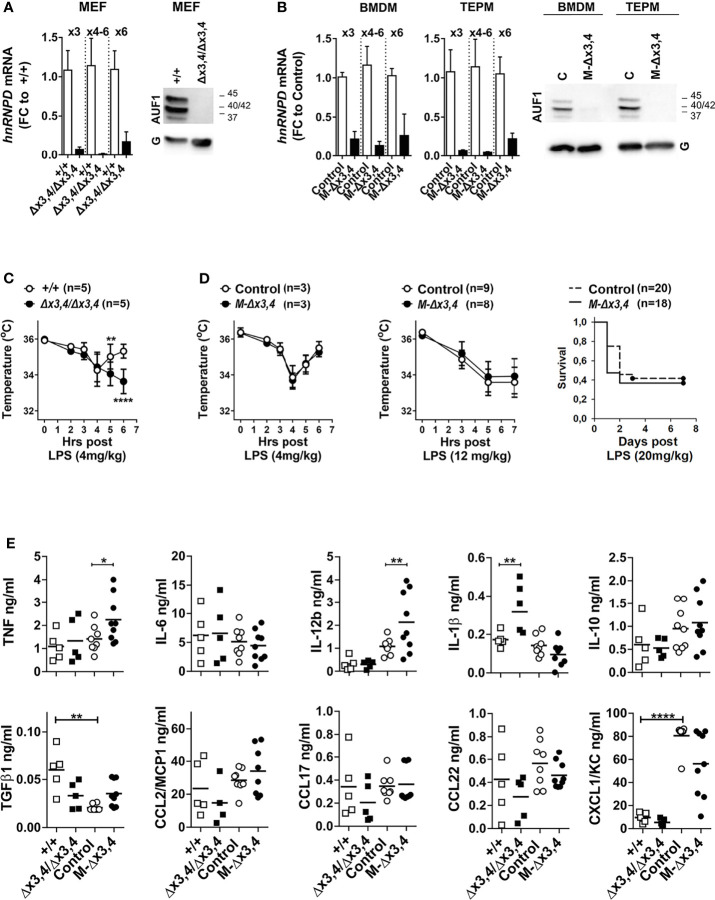
Whole-body and myeloid mutants of AUF1s differ in their responses to endotoxemia. Detection of *hnRNPD* mRNAs containing exons (x) 3 to 6 *via* qRT-PCR; and of AUF1 protein isoforms *via* immunoblots in extracts from **(A)** *hnRNPD^+/+^*, and *hnRNPD^Δx3,4/Δx3,4^* mouse embryonic fibroblasts (MEFs)**;** and **(B)** bone-marrow derived (BMDM) and thioglycolate-elicited peritoneal macrophages from control and *LysMCre^+^hnRNPD^flx3,4/flx3,4^ (M-Δx3,4)* mice. Bar graphs show mean fold change values ( ± SD) relative to the corresponding controls. Representative immunoblots indicate signals detected by an anti-AUF1 antibody or GAPDH (G) for quantitation. **(C)** Body temperature measurements of control and hnRNPD*^Δx3,4/Δx3,4^* mice at the indicated time-points post the intraperitoneal administration of the indicated does of LPS. **(D)** Body temperature and Survival measurements of control and *M-Δx3,4* mice at the indicated time-points post the intraperitoneal administration of the indicated doses of LPS. Line graphs depict mean temperature values ( ± SD); or Kaplan-Maier percentile cumulative survival. n denotes number of mice per group. **(E)** Detection of inflammatory mediators in the sera from control, whole body (n=5/genotype) and myeloid mutant mice (n=8-9/genotype) 90mins post administration of 4mg/Kg LPS. Scatter plots indicate individual and mean protein values (line) as detected *via* cytometric bead arrays. For graphs in **(A, C–E)** (*,**,****) denote p values ≤ 0.05, ≤ 0.01 or 0.00001 respectively as determined *via* One-Way ANOVA.

Our original strategy entailed targeting exons 3 and 4 of the *hnRNPD* gene to debilitate the RRMs present in all four AUF1 isoforms. Mutant *hnRNPD^flx3,4/flx3,4^* mice containing functional *hnRNPD* alleles amenable to loxP-mediated recombination (**Figure S1A**), were derived *via* gene-targeting manipulations of embryonic stem cells, germline removal of antibiotic selection cassettes and inbreeding to a C57Bl6/J background for 12 generations. To generate a mutant *hnRNPD^Δx3,4^* allele, we crossed *hnRNPD^flx3,4/+^* mice to a mouse line expressing germline-active Cre recombinase. Subsequently, *hnRNPD^Δx3,4/+^* mice were intercrossed to yield homozygous *hnRNPD^Δx3,4/Δx3,4^* mice. Examination of the F2 progenies at post-natal day 10 (P10) revealed a skew in mendelian segregation relating to a 30% loss in the *hnRNPD^Δx3,4/Δx3,4^* genotype (**Figure S1B**). However, *hnRNPD^Δx3,4/Δx3,4^* embryos were properly detected till the embryonic day E14.5, suggesting that their post-natal loss was not due to early embryonic lethality. Moreover, the majority of *hnRNPD^Δx3,4/Δx3,4^* mice identified post birth displayed a delay in weight gain (**Figure S1C**), and 50% succumbed during the two months of age (**Figure S1D**). The remaining that survived to adulthood displayed fertility issues and perished progressively past the 6-months of age. The analyses of AUF1-encoding mRNAs and proteins in extracts from *hnRNPD^+/+^*, and *hnRNPD^Δx3,4/Δx3,4^* mouse embryonic fibroblasts (MEFs) indicated the near-complete loss of all isoforms (**Figure 1A**). Thus, the genetic removal of exons 3 and 4 did not yield an RRM deficient mutein but instead led to the diminished synthesis of AUF1s.

To identify whether AUF1s have specific functions in innate immune cells, we restricted the *Δx3,4* mutation in the mouse myeloid linage *via* crossing the *hnRNPD^flx3,4/flx3,4^* mice to a *LysMCre^+^* line. Contrary to whole-body mutants, *LysMCre^+^ hnRNPD^flx3,4/flx3,4^* mice (termed hereafter as *M-hnRNPD^Δx3,4^* or *M-Δx3,4* mice) appeared phenotypically normal through a 12-month observation period. As in the case of *hnRNPDΔ^x3,4/Δx3,4^* MEFs, the reduction of AUF1 proteins in *M-hnRNPD^Δx3,4^* mice was verified in bone marrow-derived (BMDMs), thioglycolate-elicited peritoneal (TEMPs) macrophages and peritoneal Gr1^+^ polymorphonuclear cells (PMNs) but not in other myeloid derivatives like e.g. Siglec-F^+^ peritoneal eosinophils (**Figure 1B** and **Figure S1E**).

Published data on obligatory AUF1 deficient mice demonstrated their increased susceptibility to the systemic administration of lipopolysaccharides (LPS; endotoxins) from Gram-negative bacteria. LPS activates the canonical TLR4 pathway in immune and non-immune cells triggering an excessive cytokine storm (15). This leads to a variety of cytokine-induced danger signals in tissues and changes in physiology. Depending on the LPS dose, such changes range from hypothermia to systemic organ failure and lethality. To test whether our *mutant* mice responded to endotoxemia in an exaggerated manner, we challenged them first with a low dose of LPS (4mg/kg) and monitored changes in body temperature. In *hnRNPD^+/+^* mice, this dose elicited a temperature drop till the 4^th^ hour post treatment which then recovered by the 6^th^ hour (**Figure 1C** **)**. Under the same conditions, the hypothermic response of *hnRNPD^Δx3,4/Δx3,4^* mice verified their enhanced susceptibility. The drop in their body temperature was not transient but continued past the 4^th^ hour leading to a moribund state which necessitated the termination of the experiment. Surprisingly, the hypothermic response of *M-hnRNPD^Δx3,4^* mice to the same treatment was as transient as in the case of their control and of *hnRNPD^+/+^* mice **(** **Figure 1D** **).** Similarly, the susceptibility of *M-hnRNPD^Δx3,4^* mice to higher doses of LPS prolonging hypothermia (12mg/kg) or leading to sub-lethality (20mg/kg) was comparable to that of their corresponding controls (**Figure 1D**).

The different response of the whole-body to the myeloid mutants could reflect changes in the spectrum of inflammatory mediators elicited by LPS. To address this, we measured 10 prototypical mediators in the sera of all mice challenged with the low dose of LPS for 90mins (**Figure 1E**). Eight of these mediators (TNF, IL6, IL12b, IL1β, IL10, and the chemokines CCL2, 17&22) were detected in a similar range between the different control groups; whereas two (TGFβ1 & CXCL1) showed intergroup variations and were thus excluded from further comparisons.

In our analysis we noted selective quantitative differences between the mutants. *hnRNPD^Δx3,4/Δx3,4^* mice possessed higher pro-inflammatory IL1β, whereas this was not observed in *M-hnRNPD^Δx3,4^* mice. The secretion of IL1β is elicited by inflammasomes which can sense signals from degenerating tissues (28, 29). Given the previous foreground on AUF1s control over senescent or homeostatic programs (17), the exacerbated response of the whole-body mutants to LPS might be a secondary consequence of inflammatory tissue degenerations enhanced by the loss of such programs in afflicted tissues.

Notably, the sera from the challenged *M-hnRNPD^Δx3,4^* mice revealed augmentations in pro-inflammatory TNF and IL12b. The mRNAs encoding these cytokines are subject to ARE-mediated control, and their augmented presence connects to the enhanced LPS-response of mouse mutants lacking other ARE-BPs like *Zfp36/TTP*, *TIA1*, or *Elavl1/HuR* (4, 11). Unlike those mutants, the *M-hnRNPD^Δx3,4^* mice did not display an exacerbated response to LPS despite the presence of higher TNF and IL12b; whereas the quantity of these cytokines was not altered in *hnRNPD^Δx3,4/Δx3,4^* mice. These observations may be taken to suggest that (a) AUF1s have a more limited role upon the secretion of TNF and IL12b which may be masked by the pleiotropic events underlying the response of the whole-body knockouts, and (b) the activities of TNF and IL12b in our myeloid mutants could be compensated due to the effects of AUF1s’ loss upon other myeloid-derived effectors or inhibitors acting downstream of these cytokines.

### Myeloid AUF1 Is Dispensable for Pro-Inflammatory and Type I Immune Reactions Facilitating the Development of Chemically-Induced Intestinal Inflammation

**Figure 2:**
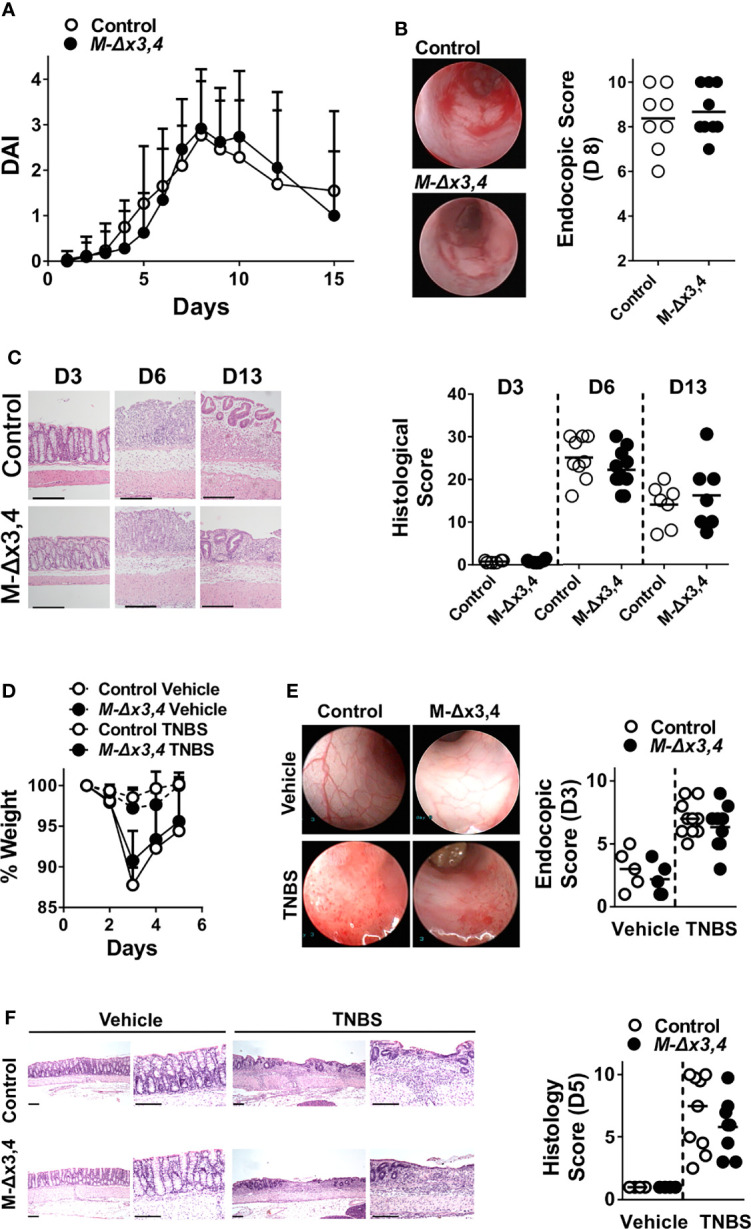
The ablation of AUF1 functions in myeloid cells does not affect the initiation nor the progression of chemically-induced intestinal inflammation. **(A)** Macroscopic Disease Activity Index (DAI) of control and *M-Δx3,4* mice after treated with DSS for seven days. Line graphs depict mean values ( ± SD; n_l_=20-28/group/genotype). **(B)** Representative photographs (left) and Scores (right) derived from the endoscopic evaluation of control and *M-Δx3,4* colons on the 8^th^ day of the DSS protocol. **(C)** *Left.* Histology of colonic tissue (left) from control and *M-Δx3,4* mice on days 3, 6 and 13 of the DSS protocol. Shown are representative photomicrographs of paraffin-embedded sections stained with H&E. Such sections were used for the evaluation of mean histological scores (*Right*) from 8-11 mice/genotype/timepoint). **(D)** Whole body-weight measurements of control and *M-Δx3,4* mice sensitized with TNBS and challenged intrarectally either with ethanol vehicle or TNBS. Line graphs depict mean weight values ( ± SD) from n=5-9 mice/group/genotype **(E)** Representative photographs (left) and Scores (right) derived from the endoscopic evaluation of control and *M-Δx3,4* colons from mice treated with ethanol vehicle (n=5/genotype) on TNBS (n=9-10/genotype) for 3 days. **(F)** *Left.* Histology of colonic tissue (left) from control and *M-Δx3,4* mice on day 5 of the TNBS protocol. Shown are representative photomicrographs of paraffin-embedded sections stained with H&E. Such sections were used for the evaluation of mean histological scores (*Right*) from Vehicle treated (n=5/genotype), on TNBS treated (n=9-10/genotype) mice. For **(B–F)**, Scatter plots indicate individual and mean protein values (line). The lack of statistical significance was tested *via* One-Way ANOVA.

The invariable, yet puzzling, systemic response of our myeloid AUF1 mutants to endotoxin prompted us to screen them for more restricted, cell-mediated and organ specific inflammatory responses. We started with two well-established models of intestinal inflammation elicited by chemical irritants. In the first model, inflammation in the colon is induced by the addition of dextran sodium sulfate (DSS) in the drinking water of mice for 7 days (30). This disrupts the colonic epithelial barrier allowing entry of commensal bacterial antigens and activation of underlying innate immune cells. The inflammatory stimulus is subsequently removed to allow for the resolution of inflammation and the restitution of the epithelium. Clinical symptoms (i.e. weight loss, diarrhea and bloody stools) were monitored daily for 15 days after the initial exposure to DSS, with endoscopic evaluation of the colon on day 8 and histological assessment on days 3, 6 and 13 representing the initiation, peak and restitution phases, respectively. The evaluation of the clinical and endoscopic parameters revealed that the disease activity of the *M-hnRNPD^Δx3,4^* mice, was indistinguishable from that in the controls (**Figures 2A, B** **)**. Similarly, the histological assessment verified that the inflammatory, degenerative and restitution phases in the afflicted colons progressed invariably between mutant and control groups (**Figure 2C**). To test whether *M-hnRNPD^Δx3,4^* mice could be differentiated *via* the cytokine variations we detected in their LPS response, we sought for disturbances in IL1β, TNF, IL6 & IL10 that are commonly engaged in systemic and mucosal inflammation. However, we failed to detect any statistical differences in the secretion of these cytokines by colonic explants isolated from mice on the 13^th^ day of the challenge (**Figure S2**). In the second model, colitis was induced by the haptenating agent 2,4,6-trinitrobenzene sulfonic acid (TNBS). This model is more complex because it relies on Type I adaptive immune responses encompassing the functions of Th1 T-helper subsets (31). Yet, innate immunity is paramount in this model as some form of clinical disease can occur even in the absence of lymphocytes. For the model, TNBS in ethanol was administered intrarectally in mice 7 days after a peripheral skin pre-sensitization, whereas a parallel group received only the ethanol vehicle (32). Weight changes were monitored for 5 days after administration of TNBS (**Figure 2D**), with parallel endoscopic evaluation on day 3 (**Figure 2E**) followed by histopathology at day 5 (**Figure 2F**). The kinetics of weight loss and recovery, the endoscopic scoring and the histological analysis of afflicted colonic tissue were nearly identical in control and *M-hnRNPD^Δx3,4^* mice (**Figure 2E**).

Collectively our data indicate that myeloid AUF1s are dispensable for the proinflammatory and Type I cell-mediated immune reactions which facilitate intestinal inflammation in models of chemically-induced colitis.

### Myeloid AUF1 Is Required for the Development of Allergic Airway Inflammation

Next, we screened for changes in Type II cell-mediated responses such as those observed in allergy and hypersensitivity reactions (33). To do so we explored an animal model of human asthma where lung inflammation is elicited post the systemic sensitization of mice to Ovalbumin (OVA) and subsequent to a local challenge with aerosolized OVA. This leads to a skewed Th2 response, the production of OVA-specific IgE, the elicitation of eosinophilic lung inflammation, and airway obstruction. In this model, immunoregulatory innate immune cells promote the recruitment of eosinophils and lymphocytes; and sustain the Th2 feedback loop while counteracting pro-inflammatory, neutrophilic and Th1 responses.

**Figure 3:**
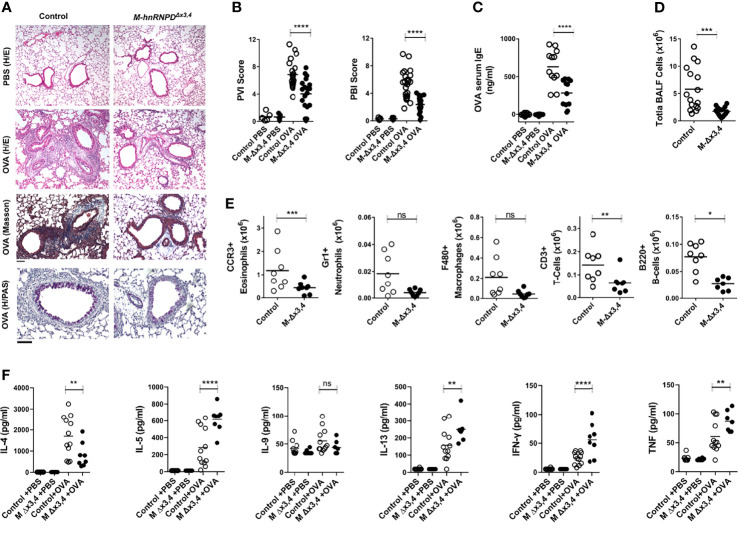
The ablation of AUF1s functions in myeloid cells attenuates the development of allergic-airway inflammation. **(A)** Representative histology of lung tissue from control and *M-Δx3,4* mice at the endpoint of treatment with aerosolized Ovalbumin (OVA). Photomicrographs of paraffin-embedded sections stained with Hematoxylin & Eosin (H&E) for the generic assessment of pathological features of peribronchial and perivascular inflammation in mice treated either with OVA or PBS vehicle; as well as Masson trichrome for fibrosis and Periodic Acid Schiff (PAS) for bronchial mucus metaplasia in OVA challenged mice. Scale bar corresponds to 50μm. **(B)** Estimation of Perivascular (PVI) and Peribronchial (PVI) Inflammation Score in Control and *M-Δx3,4* mice. Scatter plots indicate individual values and means (lines) derived from the scoring of H&E stained sections derived from n=17-22 mice/genotype. **(C)** Quantitation of total anti-OVA IgE in the sera of control and *M-Δx3,4* mice challenged with OVA or PBS vehicle. Scatter plots indicate individual values and means (lines) as assessed *via* ELISA of sera from n=11 mice/genotype. **(D)** Endpoint quantitation of total cell numbers in BALF from Control and *M-Δx3,4* mice treated with OVA. Scatter plots indicate individual values and means (lines) as assessed *via* coulter counting of BALFs from n=15-17 mice/genotype. **(E)** Absolute quantitation of distinct cellular subsets in the BALF of Control and *M-Δx3,4* mice treated with OVA. Scatter plots indicate individual values and means (lines) as assessed *via* the flow cytometric analysis of BALFs from n=8 mice/genotypeanalyzed *via* the gating strategy indicated in **Figure S3**. **(F)** Detection of T-helper lymphokines in the BALFs from control and *M-Δx3,4* mice challenged with OVA or PBS vehicle. Scatter plots indicate individual and mean protein values (line) as detected *via* cytometric bead arrays. For graphs in **(B–F)** (ns, *, **, ***, ****) denote p values > 0.05, ≤0.05, ≤0.001, ≤0.0001, ≤0.00001 respectively as determined *via* One-Way ANOVA.

Relative to mice challenged with PBS, both Control and *M-hnRNPD^Δx3,4^* mice challenged with OVA mounted the allergic response. However, the lungs of challenged *M-hnRNPD^Δx3,4^* mice displayed less peribronchial (PBI) and perivascular (PVI) inflammation, reduced fibrosis, and decreased bronchial mucus metaplasia relative to the lungs of control mice (**Figures 3A, B** **)**. This correlated with a significant reduction in OVA-specific IgE in the sera of *M-hnRNPD^Δx3,4^* mice (**Figure 3C**) and a dramatic decrease in cells infiltrating their Bronchioalveolar Fluid (BALF; **Figure 3D**). Flow cytometric analysis of BALF exudates from the *M-hnRNPD^Δx3,4^* lungs revealed that this numeric reduction was not only restricted to eosinophils but extended to all central myeloid and lymphoid infiltrates (**Figure 3E** and **Figure S3**).

The assessment of BALFs for T-helper 2 lymphokines (**Figure 3F**) revealed a significant reduction of IL-4 in the lungs of challenged *M-hnRNPD^Δx3,4^* mice whereas other lymphokines engaged in the allergic response were either augmented (e.g. IL-5, IL-13) or remained unchanged (e.g. IL-9). However, we detected significant elevations in the Th1-lymphokine IFNγ and of pro-inflammatory TNF. Together, our data indicate that the loss of AUF1s functions in myeloid cells restricts the progression of allergic airway infiltration and skews T-helper functions.

### AUF1 Is Dispensable for Macrophage Activation and Central Polarization Programs

The resistance of *M-hnRNPD^Δx3,4^* mice to allergic airway inflammation as opposed to their normotypic response to endotoxemia and colitis, suggested that in innate cells, AUF1 does not act as an anti-inflammatory factor. Instead it appears to be engaged in signal-induced programs balancing cell-mediated immune reactions.

**Figure 4:**
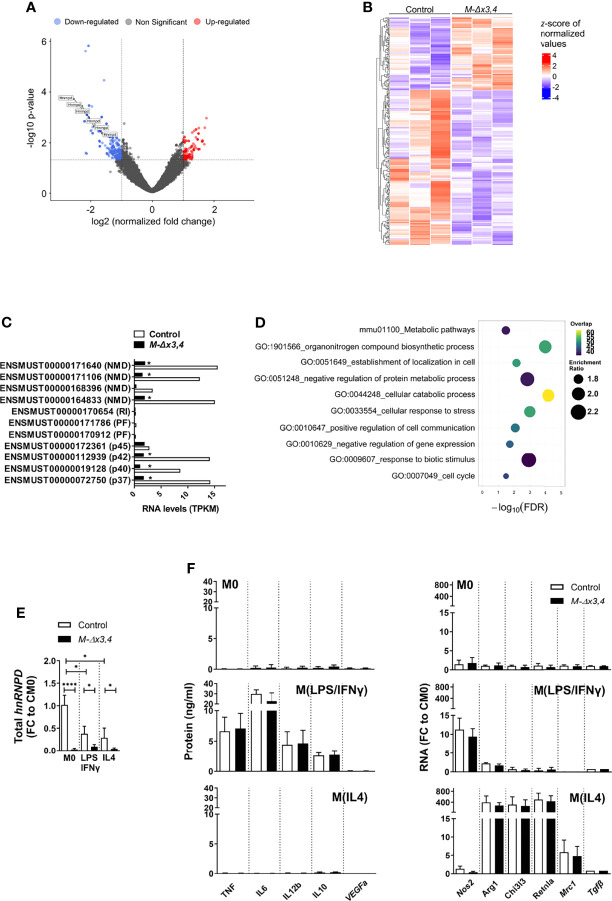
Macrophages lacking AUF1 functions display minimal changes in their activation programs and properly acquire M1- and M2a- like phenotypes *in vitro.* **(A)** Volcano plot depicting the distribution of the adjusted P values (-Log10 (adjusted P value) and the fold changes (Log2 FC) of mRNA transcripts differing between control and *M-Δx3,4* BMDMs treated with LPS for 4hrs and analyzed *via* 3’end sequencing. Significantly upregulated and downregulated transcripts are indicated by color. **(B)** Heatmap depicting the extend of significant changes in the levels of transcripts differing between 3 samples of LPS treated control and *M-Δx3,4* BMDMs. **(C)** Quantitation of *hnRNPD* RNA isoforms as derived from sequencing analyses of activated control and *M-Δx3,4* BMDMs. Bar graphs depict mean values of Transcripts Per Kilobase Million (TPKM) of ENSEMBL annotated RNAs for the p37,40,42 & 45 isoforms of AUF1; other protein coding fragments (PF); retained intron transcripts (RI); or RNAs yielded *via* NMD. (*) asterisks denote statistical significance as per the bioinformatic analyses of the datasets. **(D)** Functional classification of differential expressed transcripts as identified *via* Webgestalt (2019). Bubble plots indicate the enrichment scores and overlap of the functional categories. **(E)** Quantitation of total hnRNPD mRNA in Control and *M-Δx3,4* BMDMs (n=5) in a resting state (M0), following exposure to LPS+INFγ, or IL4. Bar graphs denote mean fold changes (FC ± SD) in exon 3 containing mRNAs relative to resting control values (CM0) as assessed *via* qRT-PCR. **(F)** Quantitation of factors marking the classical or the alternative polarization of macrophages in control and *M-Δx3,4* BMDMs (n=10) either in a resting state (M0), following exposure to LPS+IFNγ, or IL4. Bar graphs denote mean values ( ± SD) of secreted proteins assessed *via* Cytometric bead arrays; or mean fold changes (FC) in mRNAs relative to resting control values (CM0) as assessed *via* qRT-PCR. In all bar graphs, (*, ****) denote p values ≤0.05 or ≤0.00001 respectively as determined *via* unpaired Student’s t-test.

To gain further insight into this, we focused our subsequent analyses on macrophages as a prototypical innate-immune subset. Given the prior connection of AUF1s to pro-inflammatory control, we sought for comprehensive changes in RNA expression incurred by the loss of this RBP in macrophages activated by the predominant TLR4 ligand -i.e., LPS- for 4hrs. We decided to assess only mature mRNAs associated with the response without considering immature, fragmented and non-coding RNAs. To do so, we used 3′ end sequencing of transcripts (Quantseq) and performed our analysis on transcript rather than on gene level. Our search for changes (with a log2 Fold Change of 1 and p value<0.05) between the profiles of *M-hnRNPD^Δx3,4^* and control BMDMs showed an unexpectedly small number of differentially expressed transcripts (424 transcripts corresponding to 204 genes) **(** **Figure 4A** and **Table S1** **).** Moreover, 2/3 of the transcripts appeared as downregulated in AUF1-deficient macrophages **(** **Figure 4B** **),** contrasting the original hypothesis on AUF1s’ as primary decay-promoting factors. By inspecting the dataset for changes in *hnRNPD* RNAs we verified the reduction of three RNA isoforms; and in three RNAs derived from Nonsense-mediated decay (NMD) **(** **Figures 4A, C** **)**. Notably, the RNAs of other isoforms including that of the larger p45 **(** **Figure 4C** **)** were poorly expressed in macrophages suggesting the predominance of the p37, p40 & p42 isoforms in these cells.

To explore further the biological consequences of these differences in macrophage activation, we performed functional analysis using WebGestalt (2019). Ten weighted terms were identified as significant with an FDR<0.05 **(** **Figure 4D** **)**. As expected, the term “negative regulation of gene expression” was significantly enriched in the dataset and included RNA regulators involved in nuclear and cytoplasmic events besides *hnRNPD* (e.g. *Elavl1, Eif4a3, hnRNPs A2/B1* & *U, Pum1*). This implied that AUF1s may act in the apex of several regulons for RNA control. However, these regulons did not connect to changes in cytokines. Rather they connected to (a) metabolic changes such as those relating to nitrogen containing biomolecules or ATP; (b) negative effects of stress in protein modification or use and (c) extracellular signals transmitted by biotic stimuli such as those produced by the same or other cells.

We postulated that AUF1s may regulate innate immune responses depending upon signal-induced changes in cellular metabolism as in those inducing the wide spectrum of polarized macrophage phenotypes. Extremes of this spectrum can be elicited *in vitro* through different signaling combinations supporting either the classical pro-inflammatory phenotype (M1-like); or a multitude of alternative phenotypes (M2-like; M2a,b,c,d) (34). M1-like macrophages are driven by Toll-like receptor (TLR) and interferon signals and use Nitric Oxide Synthase 2 (NOS2) to metabolize arginine to nitric oxide (NO) which can be further metabolized to downstream reactive nitrogen species. Functionally, they mount pro-inflammatory type-I and Type III immune responses against bacteria, intracellular pathogens, and tumor cells and support pathologic tissue damage like in sepsis and IBD and cellular transformation if uncontrolled. Differently, the major subset of M2 macrophages is driven by IL-4 (and/or IL13; M2a) and use Arginase 1 (ARG1) to hydrolyze arginine to urea and ornithine for the support of polyamine and proline synthesis. Functionally, M2 macrophages inhibit M1-like pro-inflammatory responses, promote vascular remodeling, tissue regeneration and helminth control; but also support pathologic Type II immune responses (e.g., as in allergy), tumor vascularization and growth (35, 36).

In response to signals promoting a classical M1 phenotype (i.e., LPS+IFNγ) the total levels of *hnRNPD* transcripts were reduced (**Figure 4E**). Still, M1 polarized *M-hnRNPD^Δx3,4^* BMDMs secreted TNF, IL6, IL12, IL10 and expressed the Nos2 and several chemokine mRNAs at levels comparable to control macrophages and only under the specific signaling regime (**Figure 4F** and **Figure S4**). Intriguingly, and in response to the M2-promoting cytokine IL-4, *hnRNPD* transcripts were also reduced (**Figure 4E**); and *M-hnRNPD^Δx3,4^* BMDMs elevated properly the characteristic *Arg1, Mrc1, Retnla/Fizz1, Chi3l3/Ym1* mRNAs whereas M1 markers remained silent **(** **Figure 4F** and **Figure S4** **).**

Together, our *ex vivo* data indicate that AUF1s may not be required to establish central macrophage activation and M1 or M2a-like polarization programs. However, their connection to regulatory catabolic programs may be linked instead to their requirement for transition between programs occurring in other polarization settings.

### AUF1s Is Required for the Adenosine-Induced Transition of Pro-Inflammatory Macrophages Towards a Pro-Angiogenic Phenotype

**Figure 5:**
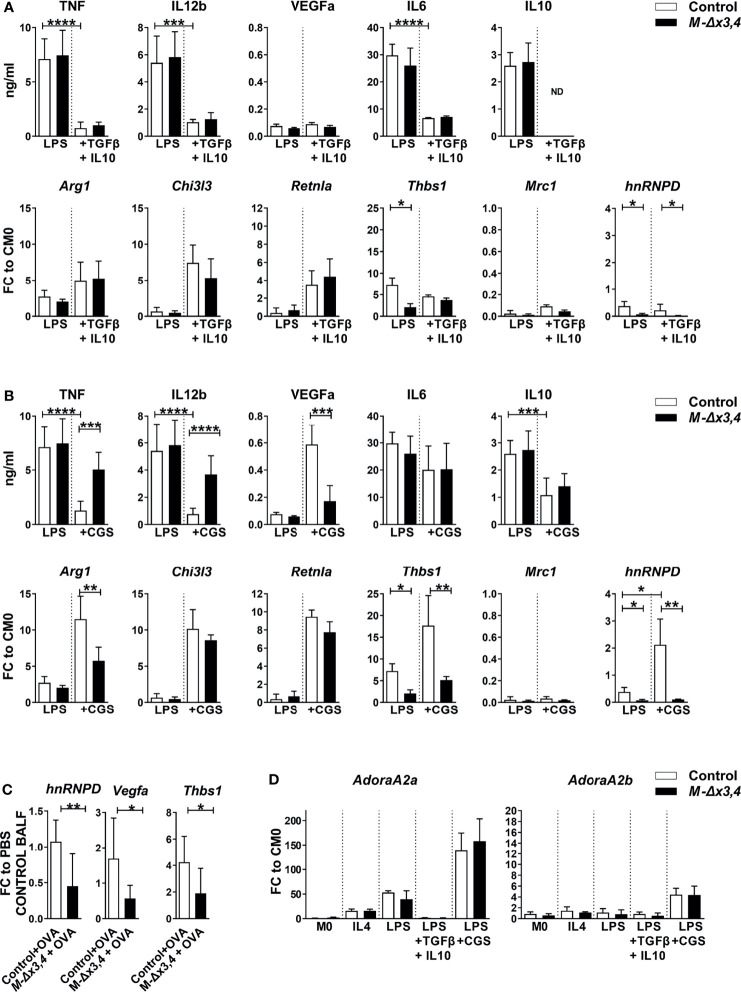
Macrophages lacking AUF1s functions show defects in their transitions in response to adenosine receptor signals but not in response to TGFβ and IL10. Quantitation of factors marking the classical or the alternative polarization of macrophages as well as detection of *hnRNPD* expression in response to **(A)** the combination of TGFβ+IL10 and; **(B)** adenosine A2A receptor agonist (CGS) in LPS-activated control and *M-hnRNPD^Δx3,4^* BMDMs (n=10). Bar graphs denote mean values ( ± SD) of secreted proteins assessed *via* Cytometric bead arrays; or mean fold changes (FC) in mRNAs relative to resting control values (CM0) as assessed *via* qRT-PCR. **(C)** Detection of *hnRNPD*, *Vegfa* and *Thbs1* mRNAs in BALF cells from OVA-treated samples (n=5-6) relative to untreated (PBS) values as assessed *via* q RT-PCR. **(D)** Quantitation of *Adora2a* and *Adora2b* mRNAs in control and *M-hnRNPD^Δx3,4^* BMDMs (n=10) in response to the indicated signals. Bar graphs denote mean values ( ± SD) or mean fold changes (FC) in mRNAs relative to resting control values (CM0) as assessed *via* qRT-PCR (*, **, ***, ****) denote p values ≤0.05, ≤0.001, ≤0.0001, ≤0.00001 respectively as determined *via* unpaired Student’s t-test.

*In vivo*, M1- and M2- like macrophages co-exist in inflammatory settings, and their plasticity allows them to switch their phenotype in response to microenvironment derived cues. Apart from the highly polarized macrophages induced by LPS ± IFNγ or IL4, several different alternative subsets have been described *in vitro.* These subsets presumably mimic phenotypes switching in response to particular tissue signals that block one phenotype for another to occur (25, 37). For example, when LPS-primed macrophages are exposed to the combination of homeostatic TGFβ and anti-inflammatory IL10 they “deactivate” proinflammatory mediators and acquire an M2-like identity (also known as M2c) (38). In response to TGFβ and IL10, the expression of *hnRNPD* mRNAs was as low as in the case of macrophages treated with LPS; still, LPS-activated *M-hnRNPD^Δx3,4^* BMDMs reduced their secretion of TNF, IL-6 and IL-12 while upregulating the M2-associated mRNAs of *Arg1, Chi3l3* and *Retnla* (**Figure 5A**) in a manner similar to control macrophages.

Another macrophage switching effect is observed, when the M1 macrophages are exposed to purinergic signals stimulated by adenosines (described loosely as M2d) (39). Adenosines bind to A2A receptors to suppress pro-inflammatory mediators while enhancing the expression of M2 markers; but also augment the expression of vascular remodeling factors like VEGFα and one of its downstream regulators, Thrombospondin-1 encoded by the *Thbs1* mRNA (40, 41). To explore the response of LPS-activated macrophages to adenosines, we used the specific adenosine A2A receptor agonist, CGS-21680 (CGS). In control BMDMs primed with LPS, CGS suppressed the expression of TNF, IL-12b and IL10 but not of M1-related chemokines; induced the secretion of VEGF and the expression *Arg1, Chil3l3, Retnla and the Thbs1* mRNAs; and -strikingly- augmented the expression of *hnRNPD* transcripts (**Figure 5B** and **Figure S5**). On the contrary, *M-hnRNPD^Δx3,4^* BMDMs exposed to LPS+CGS failed to downregulate specifically TNF and IL12 and were unable to augment properly VEGFα and the, *Thbs1* and *Arg1* mRNAs (**Figure 5B** and **Figure S5**).

The compromised expression of VEGFα & Thrombospondin as opposed to the enhanced TNF by *M-hnRNPD^Δx3,4^* BMDMs suggested that AUF1s may be involved in the macrophage specific support of vascular permeability of inflamed tissues. Indeed, we noted that the reduction of *hnRNPD* expressing cells in the BALF of OVA challenged *M-hnRNPD^Δx3,4^* mice related to comparable reductions in the *Vegfa* and *Thbs1* mRNAs (**Figure 5C**).

Three pieces of evidence suggested that AUF1s control selective transcripts rather than adenosine receptor signaling. First, the induction of *Chil3l3 and Retnla* mRNAs and the reduction of secreted IL10 in *M-hnRNPD^Δx3,4^* BMDMs were comparable to those in control macrophages (**Figure 5B**) Second, the expression of the *Adora2a* mRNA encoding the A2A receptor was enhanced 150-fold in both control and AUF1 lacking macrophages exposed LPS+CGS and 50-fold in those treated with LPS whereas it was only minimally affected by other macrophage signals (**Figure 5D**). Similarly, the loss of AUF1s did not affect the macrophage expression of *Adora2b* mRNA encoding the A2B receptor that may act synergistically to A2A and which was exclusively enhanced 5-fold by LPS+CGS (**Figure 5D**). Finally, the examination of downstream PKA activity which is commonly activated by LPS and LPS+CGS signals was not significantly altered by the loss of AUF1s (**Figure S6**).

These data indicate that AUF1s are required downstream of adenosine signalers to control specific transcripts during the switch of pro-inflammatory macrophages to those supporting vascular remodeling.

### The Loss of AUF1s in Myeloid Cells Inhibits Neo-Vascularization and Immune Infiltration of Tumors

**Figure 6:**
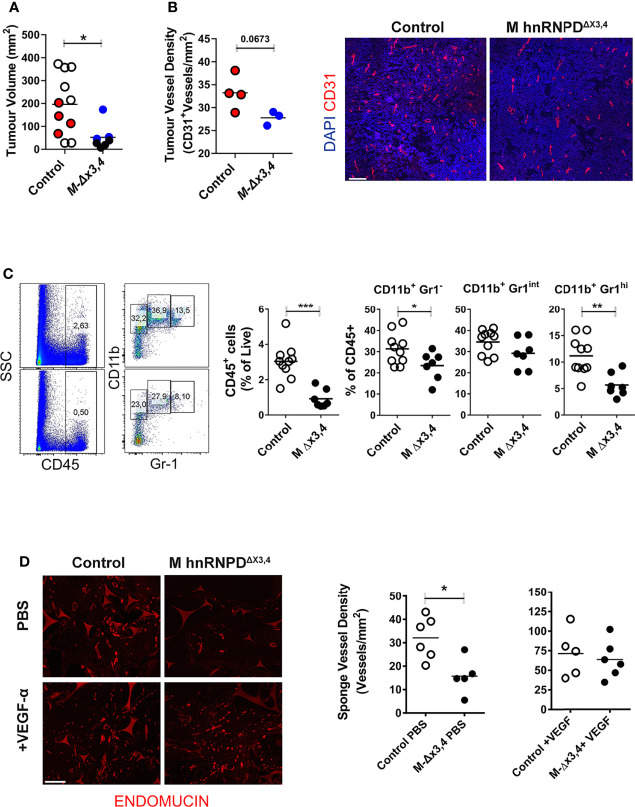
The ablation of AUF1 functions in myeloid cells attenuates tumor infiltration and the vascularization of inert grafts. **(A)** Endpoint quantitation of subcutaneous B16F0 tumor sizes of control and *M-hnRNPD^Δx3,4^* mice. Scatter plots indicate individual values and means (lines) of tumor volumes, n=10 mice/per genotype. Colored circles indicate samples that were selected for the analysis in tumor vessels density. **(B)** Graphs (left) denote mean values ( ± SD) of blood vessels per mm2 of midline section of B16 tumor following immunofluorescent staining of midline with endothelial marker CD31 and counterstained *via* DAPI as indicated in the representative photomicrographs (right). **(C)** Quantification of the percentage of live total CD45+ cells and subgated myeloid cells detected *via* CD11b/Gr-1 expression infiltrating B16-tumors of control and *M-hnRNPD^Δx3,4^* mice as analyzed *via* flow cytometry analysis. Representative dot plots (*right*) and scatter plots (*left*) depicting individual values and means (lines) from n=11 mice/per genotype. **(D)** Endomucin staining patterns of midline sections of engraphed sponges (left) and quantitation of microvessel density in sponges from control and *M-hnRNPD^Δx3,4^* mice 14 days post administration of PBS or recombinant VEGF. Graphs denote mean values ( ± SD) of blood vessels per mm2 of midline sponge section of n=6 mice/genotype. In all graphs (*, **, ***) denote p values ≤0.05, ≤0.001 or ≤0.0001 respectively as determined *via* unpaired students t-test (on **A**) or One-Way ANOVA **(B–D)**.

M2-like pro-angiogenic macrophages usually appear as tumor-associated infiltrates (TAMs) to support tumor angiogenesis and growth (42). To connect the functions of myeloid AUF1s to tumor growth we used the model of B16 melanoma tumors transplanted subcutaneously in the flanks of syngeneic mice. Contrary to tumors in control mice, the tumors in *M-hnRNPD^Δx3,4^* mice grew to smaller volumes at the endpoint (**Figure 6A**), posing difficulties in their comparative evaluation of vessel density. In an effort to gain insight on tumor vascularization, we selected whose volume was proximal to the lower range of control tumor volumes compare their vessel density as marked by endothelial marker, CD31 (PECAM). As shown in **Figure 6B**, a reducing trend in the number of CD31^+^ vessels were observed in the tumors from *M-hnRNPD^Δx3,4^* mice but their low number precluded statistical validation. To gain indirect insight we assessed the inflammatory infiltration of all tumors following dissociation and analyses of their immune infiltrates *via* flow cytometry. The percentage of total immune CD45+ tumor infiltrates in *M-hnRNPD^Δx3,4^* mice was reduced 4-fold relative those in the tumors of control mice indicating a possible defect in tumor vasculature impeding immune infiltration (**Figure 6C**). We also noted reductions amongst the myeloid CD45+ cells expressing high levels of Gr1+ which includes neutrophils and myeloid-derived suppressive cells; and on CD11b+ Gr1 negative subsets suggesting limitations in the general myeloid influx.

To get a direct answer as to whether the loss of AUF1s in myeloid cells limits their effect on blood vessels, we exploited an *in vivo* angiogenic model measuring neovascularization directly (43). In this model, inert biomaterials in a sponge-like form were engrafted subcutaneously in the flanks of mice and were injected either with PBS or recombinant VEGFα every 2 days. At the 14^th^ day sponges were excised and processed for the immunohistochemical enumeration of blood vessels using the endothelial marker endomucin. As demonstrated in **Figure 6D**, the vessel density of PBS-treated sponges engrafted in *M-hnRNPD^Δx3,4^* mice was nearly half of those in the grafts of control mice. In contrast, the VEGFα-treated sponges possessed an equally elevated blood vessel density in both the grafts in control and mutant mice.

### Divergent AUF1-Mediated Control Upon Its Macrophage Targets

**Figure 7:**
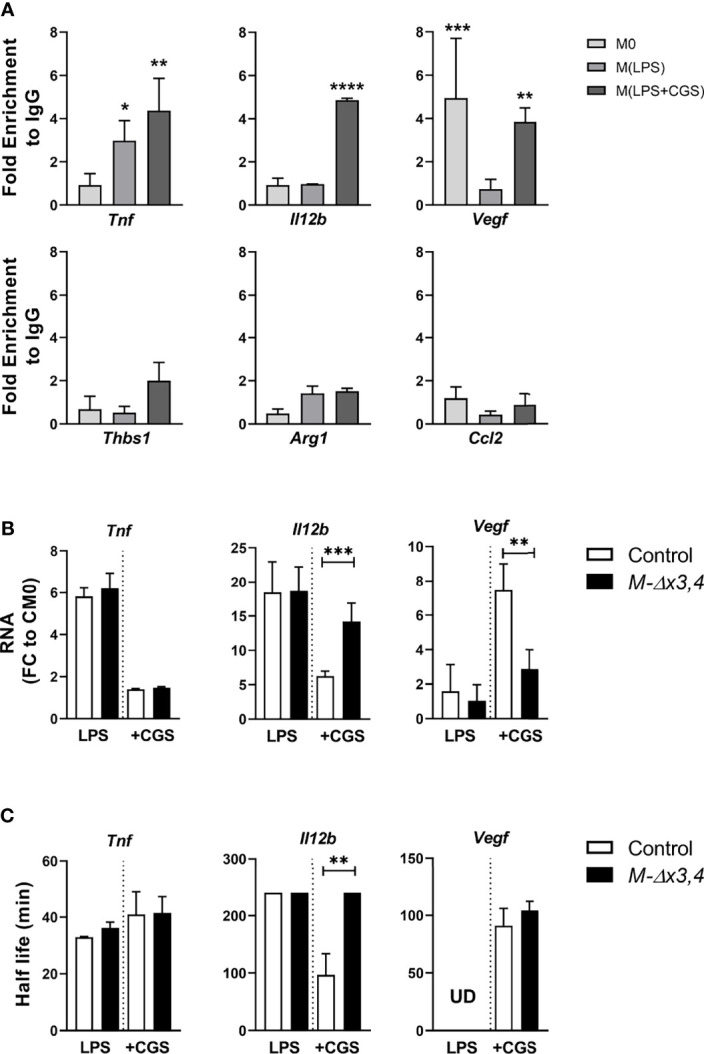
Adenosines promote the binding of AUF1 upon selective transcripts leading to their differential control. **(A)** qRT-PCR detection of selected mRNAs tested to IP with anti-AUF1 in control BMDMs activated with LPS or LPS+CGS compared to untreated (M0). Enrichment of each mRNA in AUF1-IP samples compared with its abundance in IgG-IPs and normalized to mRNA expression levels. Enrichments above two-fold were considered significant. **(B)** Expression levels of mRNAs bound by AUF1 were measured in extracts from BMDMs activated with LPS or LPS+CGS. **(C)** The half-lives (*t*_1/2_) of *Tnf, Il-12b* and *Vegf* mRNA were quantified by measuring the time required for reducing transcript levels to 50% of their original abundance after adding actinomycin as in **Figure S7B**. Graphs depict mean values ± SD. Note that half-lives in *Il-12b* mRNAs under LPS or under LPS+CGS in the mutants lack variation since they exceed monitoring times and as such were assigned a common maximal value. In all cases, data were analyzed by two tailed, unpaired Student’s t test. *p ≤ 0.05, **p ≤ 0.01 ***p ≤ 0.001, ****p ≤ 0.0001.

Our analysis on macrophages suggested that AUF1s may affect a transcript specific regulon to facilitate the functional switch from the pro-inflammatory to the pro-angiogenic state. To gain insight on how AUF1s modulate such transcripts, we focused on those that appear as most affected by the loss of AUF1s’ functions, namely the *Tnf, Il12b, Vegfa and Thbs1* mRNAs (44). First, we immunoprecipitated AUF1-containing RNPs (RIP) from cytoplasmic extracts of resting, LPS or LPS+CGS treated BMDMs for 4hrs using a specific antibody recognizing all four AUF1 isoforms. To estimate background, we performed parallel immunoprecipitations with an isotype-matched antibody. The efficiency of the RIP was monitored using immunoblots for the AUF1 proteins as antibody-bound (e.g. **Figure S7A**). Subsequently, the RNA content of the RNPs was analyzed *via* qRT-PCR and values were normalized to the levels of expression of each mRNA (**Figure 7A**). Enrichments above 2-fold were considered as significant. This analysis demonstrated that AUF1:RNPs associate only with the *Vegfa* mRNA in resting macrophages; with the *Tnf* mRNA in LPS stimulated macrophages; and most strongly with the *Il12b*, *Vegfa* and *Tnf* mRNAs in adenosine-stimulated macrophages. In contrast we did not observe associations with the *Ccl2* mRNA which we used as a relevant control; neither with the *Thbs1* mRNA indicating that its reduction could be due to the loss of eliciting VEGF (45).

Further comparison of the levels of these mRNAs in extracts from Control BMDMs revealed that CGS suppressed the LPS-induced expression of the *Il12b* mRNA whilst augmenting that of the *Vegfa* mRNA; yet it failed do so in *M-hnRNPD^Δx3,4^* macrophages (**Figure 7B**). These data correlated to the differences we observed in the secretion of IL12b and VEGF by these cells **(** **Figure 5B** **)**. Notably, and in contrast to its protein (**Figure 5B**), the *Tnf* mRNA was properly suppressed by CGS in both control and *M-hnRNPD^Δx3,4^* BMDMs (**Figure 7B**). We postulate that AUF1s either control the translation of the *Tnf* mRNA or the post-translational release of the TNF protein.

The analysis of the decay of these mRNAs following the arrest of their transcription by Actinomycin D was most revealing (**Figures 7C**, **S7B**). CGS had a potent destabilizing effect upon the *il12b* mRNA which was ablated in the absence of AUF1s. In contrast the decay of the *Vegfa* mRNA was not affected by the loss of AUF1s suggesting that AUF1s are required for its nuclear synthesis as promoted by CGS. Finally, the decay of the *Tnf* mRNA was not affected neither by the loss of AUF1s nor by CGS. Taken together these data indicate that AUF1s are involved in various transcript specific RNPs that respond to adenosine signaling which independently promote mRNA decay (e.g. *Il12b* mRNA), nuclear synthesis (e.g. *Vegfa* mRNA) or translation (e.g. *Tnf* mRNA).

## Discussion

In this study, we sought for changes to inflammatory responses caused by the loss of AUF1s in innate immune cells. To abrogate the functions of all protein-coding AUF1 isoforms in the mouse, we used gene targeting to render two exons 3 and 4 of the murine *hnRNPD* gene as conditionally removable. Hypothetically, this removal could lead to splicing events joining exons 1 or 2 to exon 5 and the synthesis of RRM-inactive AUF1 muteins. Instead, removing exons 3 and 4 reduced the expression of all coding RNA and proteins to near undetectable values. This effect was not due to aberrations imposed by our targeting strategy on the *hnRNPD* locus since mice carrying the non-recombined, loxP-flanked, alleles expressed the AUF1 proteins appropriately. Instead, we hypothesize that the diminution of *hnRNPD* transcripts could result from their decay as incorrectly spliced variants similar to *hnRNPD* variants degraded by Nonsense-mediated-decay (46).

The gross phenotypic characteristics of our germline *hnRNPD^Δx3,4^* mice resembled those of whole body *hnRNPD* null mice; as did their enhanced sensitivity to endotoxemia. In contrast, in mice where *Δx3,4* mutation was restricted in descendants of the myeloid lineage and mostly monocytes, macrophages, and polymorphonuclear cells did not phenocopy the endotoxic response of whole-body mutants. It is, therefore, likely that the enhanced endotoxic response of whole-body mutants does not pertain to dysfunctions within inflammatory cells but rather from enhanced tissue degenerations imposed by AUF1’s control over cellular senescence or development (17). This is supported further by the high levels of serum IL1β observed in endotoxic *hnRNPD^Δx3,4^* and *hnRNPD* null mice [**Figure 1E** & (17)]. The secretion of IL1β is elicited *via* inflammasomes which are in turn activated by danger and senescent signals. Moreover, the activation of inflammasomes can support a specialized form of cell death called pyroptosis which can feedback to enhance the damaging response (28, 29).

In our analysis, we did not find any evidence for impediments in the differentiation of macrophages and polymorphonuclear cells in the myeloid mutants of AUF1s. Although we cannot exclude such a possibility, the proper response of these mice to endotoxemia, which requires emergency hematopoiesis (47, 48) supports the notion that AUF1s may not be essential for the ontogeny of myeloid descendants. On the other hand, our analyses on the sera of LPS-challenged myeloid mutants revealed counteracting augmentations (e.g. in pro-inflammatory TNF & IL12b that could result in altered cellular reactivities. To reveal such activities, we selected to challenge myeloid mutants for Type I or Type II cell-mediated reactions controlled either by pro-inflammatory or immunoregulatory circuits. For the assessment of pro-inflammatory and Type I cellular reactions we chose challenges inducing intestinal inflammation. The lamina propria underlying the intestinal epithelium contains many phagocytic macrophages supporting local homeostasis and microbiome balance (49). In Inflammatory Bowel Diseases (IBD), neutrophils and monocytes/macrophages are massively recruited from the blood to the lamina propria. Neutrophils have differential roles in IBD (50); whereas macrophages become polarized towards an “M1-like” state which uncontrollably release inflammatory cytokines, promote intestinal barrier damage, enhance Th1 and Th17 T-cell responses (51) and cannot be counteracted by anti-inflammatory “M2-like” macrophages expressing and responding to IL10 and TGFβ or primed by regulatory T-cell subsets (52). Currently, there is sufficient evidence on the dysfunction of “ARE-binding proteins” in supporting IBD (30). Moreover, a connection between mutations in the human *hnRNPD* gene and Crohn’s Disease has been revealed from studies on monozygotic twins (53). In light of the above, the indifferent response of the myeloid AUF1 mutants to DSS and TNBS induced colitis was exceptionally noteworthy. These results should be interpreted cautiously concerning human disease since these chemical models mimic only some of its aspects. However, they provide first proof of the dispensability of AUF1s in the functioning of proinflammatory macrophages in chronic intestinal inflammation.

The combination of our data on endotoxemia and colitis suggests that AUF1s have redundant roles in the activation and cytokine-induced deactivation of classical “M1-like” macrophages. This redundancy was also apparent in our *ex vivo* assays. Our holistic analyses on LPS stimulated macrophages revealed only specialized changes in mRNAs devoid of alterations in cytokine mRNAs. Moreover, the loss of AUF1 functions did not debilitate the end polarization of “M1-like” macrophages *via* LPS and IFNγ; neither did their transition towards a deactivated “M2c-like” phenotypes by IL10 and TGFβ. These contradict earlier studies in human and mouse cell lines pointing to AUF1s control over the use of pro-inflammatory mRNAs by these signals. We hypothesize that methodological differences and the use of transformed cellular settings account for this contradiction.

Unlike the responses in endotoxemia and intestinal inflammation, allergic airway inflammation entails more complex innate immune reactivities. Several studies demonstrated a positive correlation between the presence of macrophages and enhanced eosinophilic inflammation using allergic models in mouse and humans (54, 55). These studies have already revealed the active role of alternatively activated macrophages -particularly those induced by IL4 and/or IL13 - in the exacerbation of Th2/Th9-driven inflammation (56). The fact that myeloid AUF1 mutants display a strong reduction in allergic airway inflammation and IL4 in their BALF could connect to the loss of such “M2-like” functionalities. To that end, we were surprised to find that cultured macrophages lacking AUF1 could be properly polarized towards an “M2a-like” phenotype by IL-4. Arguably, the augmented presence of IFNγ in the BALF of allergen challenged myeloid AUF1 mutants could connect to an enhanced Th1 response supported by innate cells expressing high levels of IL12. These responses could have an opposing effect upon eosinophilic inflammation favoring neutrophil infiltration. However, such a switch was not detected in our mutants. Instead, all types of infiltrates were diminished in the allergen exposed myeloid AUF1 mutants. Such a phenotype could arise from aberrations in vascular remodeling, as exemplified in allergen exposed mice treated with anti-VEGF antibodies (57). Increased expression of VEGF is a common feature in the BALF of asthmatic patients, whereas its overexpression in the murine lung induces an asthma-like phenotype with features including vascular remodeling, mucus metaplasia, and augmented Th2 inflammation. VEGF is produced by infiltrating cells and the resident tissue, which includes alveolar macrophages and required for the infiltration of inflammatory cells in allergic airway inflammation (58).

The reduced number of VEGF-expressing infiltrates in the BALF of our myeloid mutants posits that their defective infiltrating response to allergen relates to defects in vascular permeability promoted by specialized alternative macrophage subsets. Since our focus was on immunocellular events, we did not look for changes in the responses of lung endothelia. However, our hypothesis was further supported by our tumor data, where the positive effect of M2-like, pro-angiogenic tumor-associated macrophages (TAMs) is well established (59). The myeloid loss of AUF1 compromised the growth of transplanted B16 melanomas correlating with reduced infiltration of TAMs (and TILs; data not shown)-despite the fact that tumor cells can produce part of the VEGF required for their vascularization. The poor growth of tumors in the setting of myeloid AUF1 deficiency prohibited the proper statistical evaluation of impediments in their vascularization or remodeling. However, these became apparent in the myeloid mutants failing to vascularize an inert graft; and the rescuing response of exogenously added VEGF. Moreover, we hypothesize that the defective VEGF response of myeloid-AUF1 mutants may also connect to their invariable susceptibility to LPS despite the presence of high TNF and IL12b. Vascular permeability and leakiness are critical events in endotoxemia, and VEGF blockade has a beneficial effect against it (60, 61).

Our *in vivo* observations correlate with the inability of AUF1 lacking macrophages to switch towards a pro-angiogenic phenotype (denoted by some as M2d) at least by adenosine receptor signals. Extracellularly, adenosines are generated by the surface enzymes CD73 and CD39. They transmit their signals through the surface G-coupled A2AR receptors and the cAMP/CEBP pathway (62). The importance of CD73 and CD39 activities against lung injury and for tumor growth is well documented. The lack of these enzymes -and hence of adenosines- provides susceptibility to allergic airway inflammation (63) and adenosine signaling has emerged as therapeutic target (64, 65). Concerning their functions in macrophages, adenosines were originally identified anti-inflammatory agents. It was later shown that they can promote the switch of activated macrophages towards an angiogenic phenotype expressing VEGF and independently of IL4 signaling (40), hence connecting to the *in vivo* functions of adenosine-generating enzymes. Our study connects the deranged activities of adenosine-induced pro-angiogenic macrophages incurred by the loss of AUF1s to the susceptibility of the mutant mice to airway and tumor infiltration. The full characterization of such cells in a disease setting and at a single cell level is currently lacking. However, their future identification holds promise for their exploitation in disease treatment *via* cellular ablation or addition strategies.

In macrophages, the expression of AUF1s was augmented by the agonistic activation of A2AR; and is needed to suppress TNF and IL12 whilst supporting the *de novo* expression of VEGF and Thrombospondin indicating the complex involvement of AUF1-controlled regulons in the pro-angiogenic switch. Notably, other “M2-like” mRNAs (e.g. *Il10*, *Retnla, Chi3l3*) were regulated by A2AR signals but independently of AUF1s. Moreover, TLRs and A2ARs signals remained unaffected by the loss of AUF1s. These suggested that AUF1s act in a transcript-selective fashion and downstream of A2AR signaling. Indeed, and to the limits of our analysis on pre-defined marker RNAs, only the *Tnf*, *Vegfa* and *Il12b* mRNAs were identified to associate with AUF1-containing ribonucleocomplexes. Most intriguingly, the type of regulation imposed by AUF1 on these mRNAs indicated three different types of regulons for RNA control: activation of mRNA biogenesis (*Vegfa*), activation of protein synthesis or release (*Tnf*) and mRNA degradation (*Il12b*). Given the pleiotropy AUF1s molecular activities on RNAs this is not so unprecedented. However, the confinement of its activities in selective cellular subsets -like in the case of pro-angiogenic macrophages- is noteworthy; especially considering propositions on its functional overlap with other factors (e.g. *Zfp36/TTP* and *Elavl1/HuR*) whose immune mutations have more profound effects (4, 11). Our genomic data revealed changes in RBP transcripts affected by the loss of AUF1s. Such changes could have functional consequences in the control of AUF1-regulons; or compensate for AUF1s loss in specific cellular contexts. Irrespective however of the cross-relationships affecting RNAs, the paradigm of AUF1 indicates that RBPs may control the selective diversification of cellular subtypes rather than affect RNA use in all.

## Materials and Methods

### Mice and Study Approvals

The targeting of the *hnRNPD* locus in murine ES cells was performed by Regeneron Pharmaceuticals using their proprietary *Velocigene recombineering* technology (66). Modifications included the flanking of exons 3 and 4 with loxP sites; the inclusion of and FRT-flanked neomycin resistance gene for selection proximal to the loxP of exon 3. These ES cells were integrated onto blastocysts from C57Bl6/J mice. Derivative mice where then crossed to mice expressing a germline encoded Flipase gene to remove the neomycin cassette and yield mice containing the *hnRNPD^flx3,4^* allele. To generate a mutant hnRNPD^Δx3,4^ allele, hnRNPD^flx3,4/+^ mice were crossed to a mouse line expressing germline-active Cre recombinase (67). Subsequently, hnRNPD^Δx3,4/+^ mice were intercrossed to yield homozygous hnRNPD^Δx3,4/Δx3,4^ mice. For the myeloid restriction of the mutation *hnRNPD^flx3,4/flx3,4^* mice were crossed to the *LysM-MCre^+^* line. All mouse lines were maintained in a C57BL/6J background and in the animal facilities of the Biomedical Sciences Research Center (BSRC) “Alexander Fleming” under specific-pathogen free conditions. All experiments were performed with 8 to 16 weeks old mice and in accordance to the recommendations of the BSRC’s Institutional Committee for Protocol Evaluation, the Veterinary Authorities of the Prefecture of Attika, the national legislation and the European Union Directive 63/2010. Protocols were approved by the Veterinary Authorities of the Prefecture of Attika (licenses # 4376/2014, 262781-22/04/2020, 26497-13/01/2020, 6197-21/11/2017, 4401-5/7/2016, 2823-13/06/2018, 531-13/2/2019, 26488-13/01/2020).

### LPS-Induced Endotoxemia

Mice 6- to 8-week-old were injected intraperitoneally (i.p.) with the indicated amount of LPS (LPS from *Salmonella enterica* serotype enteritidis; Sigma-Aldich, L6011). Core body temperature and survival were monitored through a period of 7 days to determine mice sensitivity to endotoxemia. For measurement of inflammatory mediators, *hnRNPD^Δx3,4/Δx3,4^* and *hnRNPD^Δx3,4^* mice were injected i.p. with LPS at 4mg/kg of body weight. Sera were collected 90 minutes later by cardiac puncture and cytokines were quantified using LEGENDplex™ Mouse Th Macrophage Panel (Biolegend, Cat. No.740846).

### DSS Induced Colitis

Mice 6- to 8-week-old were fed ad libitum for one cycle with water containing 2% (wt/vol) DSS (MW 40,000 kDa; MP Biomedicals Inc.) for 7 days followed by 8 days with regular water without DSS (68). Mice were monitored daily for changes in body weight, stool consistency and rectal bleeding. These values were used to calculate the Disease Activity Index (DAI). Endoscopy was performed using Coloview endoscopic system (Karl Storz, Germany) at day 8. Colonic inflammation was scored according to MEICS (Murine Endoscopic Index of Colitis Severity) as described (69). Mice were sacrificed at indicated time points or at the end of the protocol for the isolation of colonic tissue. Histological scoring was performed on hematoxylin and eosin (H&E) stained colon sections as previously described (70). Scores of inflammation and epithelial damage were summed to produce the overall histological score. All histological assessments were performed in a blinded fashion, by two independent investigators.

### TNBS Induced Colitis

Mice 6- to 8-week-old were pre-sensitized applying TNBS solution (1% w/v in acetone and olive oil solution 4:1) epidermally at day -7. On day 0, mice were inoculated intra-rectally with 100μl TNBS solution (2.5% w/v in absolute ethanol). Weight loss was daily monitored from day 0 to 4. Endoscopy was performed the 3rd day when severe symptoms peaked as described above (32).

### Allergic Airway Inflammation

Airway inflammation was induced by ovalbumin (OVA-sensitization) as previously described (54). Briefly, sex matched mice were pre-sensitized on days 0 and 15 *via* intraperitoneal injection of 10μg chicken egg-derived OVA (Sigma Aldich, A5503) mixed with 1mg of aluminum hydroxide (Alum) as an adjuvant dissolved in 300 μl phosphate buffered saline (PBS) on day 0 and 15. Subsequently, mice inhaled 1% OVA aerosol for 30 min/day from day 21 to 25. At the end point, bronchoalveolar lavage fluid (BALF) was collected from the lungs by gently washing airway lumina with 0.5 mM EDTA in PBS *via* a tracheal cannula. BALF cells were analyzed by flow cytometry (strategy of analysis is presented in **Figure S3**), while supernatants were used for cytokines analysis using the LEGENDplex™ Mouse Th Cytokine Panel (Biolegend, 741044). To determine the effectiveness of the immunization, anti-OVA IgE levels were measured in the serum of PBS and OVA challenged mice using Anti-Ovalbumin IgE Elisa Kit (Cayman, 500840), according to the manufacturer’s instructions. Lungs were collected, fixed in 10% formalin (VWR, 9713.500) and proceeded for histopathological examination. General morphology and inflammation were examined in H&E, Periodic acid Schiff (PAS)-H&E, and Trichrome Masson stained lung sections. Perivascular (PVI) and peribronchiolar (PBI) inflammation were assessed using a four-tier score system (0=none, 1=mild, 2=moderate, 3=severe, 4=tissue destruction) with an increment of 0.5 if the inflammation fell between two integers.

### B16 Tumor Model

Mice were injected subcutaneously in the flank with 10^6^ B16-F0 melanoma cells in 100ul PBS. Tumors were grown for 14 days. Post-mortem volume was determined using all 3 dimensions (m1×m2×m3×π/6). To prepare Single cell suspensions, tumor tissues were gently removed, cut into small pieces and incubated with 50 μg/ml Collagenase/Dispase (Roche, 10269638001) and 200 Units/ml DNAse I (Sigma-Aldrich, D5025) in RPMI 1640 (Gibco, 31870025) for 30 min at 37°C. Enzymatic dissociation was stopped with 10% FBS. Cells were washed with PBS and stained with the anti-mCD45, anti-mCD11, anti-mGr1, anti-mCD3 and anti-CD45R/B220 flow cytometry antibodies to identify immune subsets infiltrated in tumors. FACS analysis strategy is presented in Fig 6B.

### Subcutaneous Angiogenesis Assay

Growth factor induced angiogenesis assay was performed as described previously (43). Briefly, subcutaneously implanted sponges of 1 cm^3^ size were injected with 100μl of either PBS alone (as negative control) or PBS containing 10 ng/ml^-1^ VEGF (VEGF 164, R&D,493-MV), every 2 days. Sponges were excised, and paraffin embedded after 14 days.

### Histological Analysis and Immunohistochemistry

For immunohistochemistry, all tissues were embedded in paraffin blocks. Thick sections (4μm) of embedded colon and lung tissues were stained with H&E. PAS staining and Masson trichrome were performed with standard protocols to determine fibrosis and mucus secretion in lung tissues. Blood vessels were identified by immunofluorescence staining using primary antibody against Endomucin antibody [santa cruz, sc65498/V.7C7.1] followed by secondary anti-rat 546 Alexa (A11081) and vessel density was calculated as the number of blood vessels per mm^2^ of sponge section area using ImageJ software. Representative images were obtained on a Nikon ECLIPSE E200 microscope with a Nikon Digital Sight DS-5M digital camera.

### Cell Isolation and Culture

Bone Marrow Derived Macrophages (BMDMs) were isolated from tibiae and femora of 6-8 week-old mice. Isolation was performed as previously described by Warren and Vogel (1985) and then differentiated to Bone Marrow Derived Macrophages in RPMI 1640 medium (Gibco, 31870-025) supplemented with 5% FBS (Gibco, 31870-025), 1% penicillin/streptomycin (Gibco, 15140122), 1% L-glutamine (Gibco, 25030024) and 10% L-medium (produced by L929 cells) incubated for 10 days at 37°C with 5% CO_2._ Exudate macrophages, neutrophils and eosinophils were isolated from mice 48 or 72 hours upon intraperitoneal injection of 1 ml Brewer thioglycollate medium (4%; Becton-Dickinson).

### Cell Treatments

BMDMs were activated with LPS (100 ng/ml, *Salmonella* enterica *enteritidis*; Sigma L6011), IFN-γ (10 ng/ml, Peprotech 315-05), IL-4 (10ng/ml, Peprotech 214-14), CGS (10μM, Sigma, 21680), IL-10 (10ng/ml, Peprotech, 210-10) and/or TGFβ1 (10 ng/ml, Cell-signaling, 231LC) for 24 hours.

### Flow Cytometry Analysis and Sorting

For surface Single cell suspensions were prepared from either *in vivo* or *in vitro* experiments. For surface antigens, staining was performed using standard procedures. Antibodies were from BD Biosciences CD11b (M1/70); CD34 (RAM34); CD4 (H129.19); CD8 (H129.19), siclecF (E50-2440); eBioscience (F4/80), (CD16/32 (93), ScaI (D7), Biolegend GR1 (RB6-865), CD11C (N418); CD3e (145-2C11), CD206 (CO68C2), CCR3 (144505), Zombie NIR; B220 (RA3-6B2), Lin (mouse lineage panel, BD Pharmigen-559971). Cell viability was assessed by Propidium Iodide Staining. Cells were detected using FACSCantoII flow cytometer. Data were analyzed using FACSDIVA (V.6; BD Biosciences) and V10; Treestar, FlowJo).

### Immunoblotting and ELISAs

The cells were lysed in ice-cold lysis buffer (50 mM Tris-HCl, pH 7.4, 150 mM NaCl, 1 mM EDTA, 0.25% SDS, 10% Glycerol, 1% NP-40 and one tablet of complete protease inhibitors) for 15 min on ice. Lysates were analyzed on SDS-polyacrylamide gels (SDS-PAGE; 12-14%), along with protein molecular weight markers (Nippon MWP03, Fermentas, Thermo Scientific, SM0431) and blotted onto nitrocellulose membrane (GE Healthcare). After blocking with 5% milk in TBS-Tween 20 buffer, membranes were incubated with primary and HRP-conjugated secondary antibodies (Biotech, anti-rabbit HRP 1030-05 or anti-mouse HRP 4050-05) signals were visualized by enhanced chemiluminescence (ECL, GE Healthcare) using films or a ChemiDocTMXRS+ System with Image LabTM software. Primary antibodies used: anti-AUF1 (milipore, 07-260) and anti-GAPDH (Ambion, 6C5). Cell culture Supernatants or serum were analyzed *via* specific ELISA kits of TNFa, IL-10, IL-6, VEGF, IL12 (Peprotech 900-K54, 900-K53, 900-K50, 900-K99), IL-1b (Thermo-Fisher, 88-7013-88), Anti-Ovalbumin IgE (Cayman 500840) or cytometric bead-based assay panels following manufacturer’s instructions Legendplex Panels (Biolegend, Cat. No.741044 and 740846). Protein levels were normalized to cell number using crystal violet staining.

### Ribonucleoprotein Immunoprecipitation

BMDMs were stimulated with LPS or LPS and CGS for 4 hours. Cells were harvested and lysed in 300μl lysis buffer containing 100 mM KCl, 25 mM EDTA, 5 mM MgCl2, 10 mM HEPES, 0.5% NP40, 2 mM DTT, 0.2%, protease inhibitors cocktails, Vanadyl Ribonucleoside Complex (Invitrogen) and 100 U/ml RNAse OUT (Invitrogen). A small quantity of samples was kept aside as Input (total cytosolic) for Western Blot analysis and for total cytosolic RNA (RNA-input). Antibody uncoated beads (protein G), (Dynabeads™ Protein G Immunoprecipitation kit, ThermoFisher), were washed and maintained in 600 µl of NT-2 buffer (50 mM Tris, pH 7.4, 150 mM NaCl, 1 mM MgCl2, and 0.05% NP40). For IP, 400 µl of cell lysate were loaded onto the beads and incubated overnight on a rotary at 4°C. Subsequently, beads were washed four times with NT-2 buffer, one of which contained 0.7M urea and finally resuspended in NT2 buffer. Fifteen microliters of the total volume of samples were removed for immunoblot verification while the remaining volume was used for RNA isolation *via* TRI reagent. For immunoblotting, polyclonal anti-AUF1 antibody (Millipore 07-260) and a IP conformation-specific secondary antibody (anti-mouse IgG -HRP-conjugated Veriblot for IP Detection Reagent, Abcam, ab131366) were used.

### RNA Stability Assay

Stability of the mRNAs of interest was assessed by the addition of RNA polymerase II inhibitor, actinomycin D (10 μg/ml, Sigma) into cell culture for a period of 240 min. Total RNA was isolated at the indicated time points using TRI reagent and quantified using qRT-PCR. The half-live of transcripts was calculated using a fitted exponential curve. High undetermined values are arbitrarily defined.

### RNA Isolation and Analysis

Total RNAs were extracted from cells using TRI Reagent (MRC, TR 118) according to the manufacturer’s protocol. For cDNA synthesis 0.5-2μg of total RNA was reverse transcribed using MMLV-RT (Promega, M1705) and Oligo dT (NEB, S1316S) at 37°C for 1h. qRT-PCR was performed using EvaGreen SsoFast mix (Bio-Rad, Hercules, CA, USA, 172-5201) on a RotorGene 6000 machine (Corbett Research, Qiagen). Relative mRNA expression was normalized to β2-microglobulin (B2M) and calculated as the difference between the test values and the control values assigned as 1, using Bio-Rad RelQuant (Bio-Rad). Primers are listed in **Supplementary Materials.**

### Sequencing Analysis and Bioinformatics

RNA Total RNA in expansion phase was collected using TRI Reagent (Invitrogen, Life technologies). RNA was extracted according to the manufacturer’s instructions and resuspended in 30μls of nuclease free water. Total RNA was quantitated using the NanoDrop ND1000 Spectrophotometer. Samples were diluted accordingly to a mean concentration of approximately 150 ng/μl, on an area of 235.0 and their quality assessed on a Bioanalyzer (Agilent Technologies) using the Agilent RNA 6000 Nano Kit reagents and protocol (Agilent Technologies). Sequencing libraries were prepared by using the QuantSeq 3’ mRNA-Seq Library Prep Kit FWD (QuantSeq-LEXOGEN™, Vienna, Austria) and RNA-seq libraries were sequenced on an Ion Proton PI™ V2 chips, an IIon Proton™ System. Library preparation and alignment strategies are provided in **Supplementary Material**. Mapped sequences Quant-Seq BAM files were analyzed on a transcript level base with the Bioconductor package metaseqR2 (71) v.1.3.14 which has built-in support for Quant-Seq data. Differential expression analysis for the contrasts MΔχ3,4 versus Control was performed using DESeq2, edgeR, NOISeq, limma, NBPSeq, ABSSeq and DSS algorithms and a combined meta-analysis procedure by the PANDORA algorithm implemented in metaseqR2. 3’ UTR areas (and their corresponding transcripts) presenting a PANDORA p-value less than 0.05 and an absolute fold change (for each contrast) greater than 1 in log2 scale were considered as differentially expressed. Categorical enrichment analysis of differentially expressed transcripts was implemented with WebGestaltR6 version 0.4.4 R package. The method that was used was the Over-Representation Analysis (ORA) against the Biological Process functional database of Gene Ontology and Kyoto Encyclopedia of Genes Genomes (KEGG) pathways and the terms retrieved were identified at a significance level of FDR ≤ 0.05. Multiple testing adjustment was carried out with the Benjamini-Hochberg procedure. All data extracted from sequencing datasets and gene sets were plotted in R (v.4.1.0) using ggplot27 (v.3.3.5) package. Z-scores were calculated as a derivative of the normalized counts.

### Statistics

Statistical analysis was performed on Graphpad Prism 6.01 (Graphpad Software). Statistical significance was determined using unpaired Student’s t-test or One Way Anova when comparing three or more groups. Survival analysis was performed using Kaplan-Meier statistics. Results with a P-value 0.05 considered to be statistically significant.

## Data Availability Statement

The datasets for this study have been deposited in Gene Expression Omnibus (GEO) with the accession numbers GSE181222.

## Ethics Statement

The animal study was reviewed and approved by Veterinary Authorities of the Prefecture of Attika, Greece.

## Author Contributions

DLK and SG designed and supervised the study. Most the *in vivo* experiments were performed by NL and SG assisted by MS and MA. Tumor and vascularization assays were performed by SG and VK. Endoscopic measurements were performed by VN. Macrophage experiments were performed by SG, NL, ED and NS. RIPs were performed by MC and SG. Sequencing experiments were overviewed by PH, and bioinformatic analyses was performed by DT. Data interpretation and presentation was performed by DK, SG, and NL Manuscript was written and edited by DK and SG with support from ED. All authors contributed to the article and approved the submitted version.

## Funding

We acknowledge support of this work by projects InfrafrontierGR/Phenotypos (MIS 5002135) and Strategic Development of the Biomedical Research Institute Alexander Fleming (MIS 5002562), which are implemented under the Actions Reinforcement of the Research and Innovation Infrastructure and Action for the Strategic Development on the Research and Technological Sector respectively, funded by the Operational Programme Competitiveness, Entrepreneurship and Innovation (NSRF 2014-2020) and co-financed by Greece and the European Union (European Regional Development Fund). We also acknowledge support by ARISTEIA-I project No. 1096 PREcISE, funded by the Operational Programme Education and Lifelong Learning (NSRF 2007-2013) and co-financed by Greece and the European Union (European Social Fund). This research is also supported by Greece and the European Union (European Social Fund-ESF) through the Operational Programme «Human Resources Development, Education and Lifelong Learning 2014- 2020» (project MIS 5069605) and NSRF 2014-2020, European Regional Development Fund, Operational Programme “Competitiveness, Entrepreneurship and Innovation 2014-2020 (EPAnEK)” (MIS 5032782).

## Conflict of Interest

The authors declare that the research was conducted in the absence of any commercial or financial relationships that could be construed as a potential conflict of interest.

## Publisher’s Note

All claims expressed in this article are solely those of the authors and do not necessarily represent those of their affiliated organizations, or those of the publisher, the editors and the reviewers. Any product that may be evaluated in this article, or claim that may be made by its manufacturer, is not guaranteed or endorsed by the publisher.
