# Supplementary material for: Inactivation of AUF1 in Myeloid Cells Protects From Allergic Airway and Tumor Infiltration and Impairs the Adenosine-Induced Polarization of Pro-Angiogenic Macrophages
Source: Front Immunol. 2022 Feb 11;13:752215. doi: 10.3389/fimmu.2022.752215 (PMC8873154; doi:10.3389/fimmu.2022.752215)
Supplement: Supplementary file 1 [file DataSheet_1.docx]

***Supplementary Material***

**Inactivation of AUF1 in myeloid cells protects from allergic airway and tumor infiltration and impairs the adenosine-induced polarization of pro-angiogenic macrophages**

Sofia Gargani^1†^, Niki Lourou^1,2†^, Christina Arapatzi^1^, Dimitris Tzanos^1^, Marania Saridaki^1^, Esmeralda Dushku^2^, Margarita Chatzimike^1^, Nikos Sidiropoulos^2^, Margarita Andreadou^1^, Vassilios Ntafis^1^, Pantelis Hatzis^1^, Vasso Kostourou^1^ & Dimitris L. Kontoyiannis^1,2*^

^1^Biomedical Sciences Research Centre "Alexander Fleming", Institute of Fundamental Biomedical Research, Vari, Greece.

^2^ Department of Genetics, Development and Molecular Biology, School of Biology, Aristotle University of Thessaloniki, Thessaloniki, Greece

† These authors have contributed equally to this work

*Corresponding Author: Dimitris L. Kontoyiannis

Department of Genetics, Development and Molecular Biology, School of Biology, Aristotle University of Thessaloniki, 54124 Thessaloniki, Greece

Biomedical Sciences Research Centre "Alexander Fleming", Institute of Fundamental Biomedical Research, 34 Fleming Street, 16672 Vari, Greece.

E-mail: dkontoyiannis@bio.auth.gr, kontoyiannis@fleming.gr

Index:

1. Supplementary Figures S1-S7, pages 2-9
2. Supplementary Methods, pages 10-12
3. Supplementary References, page 12

## Supplementary Figures


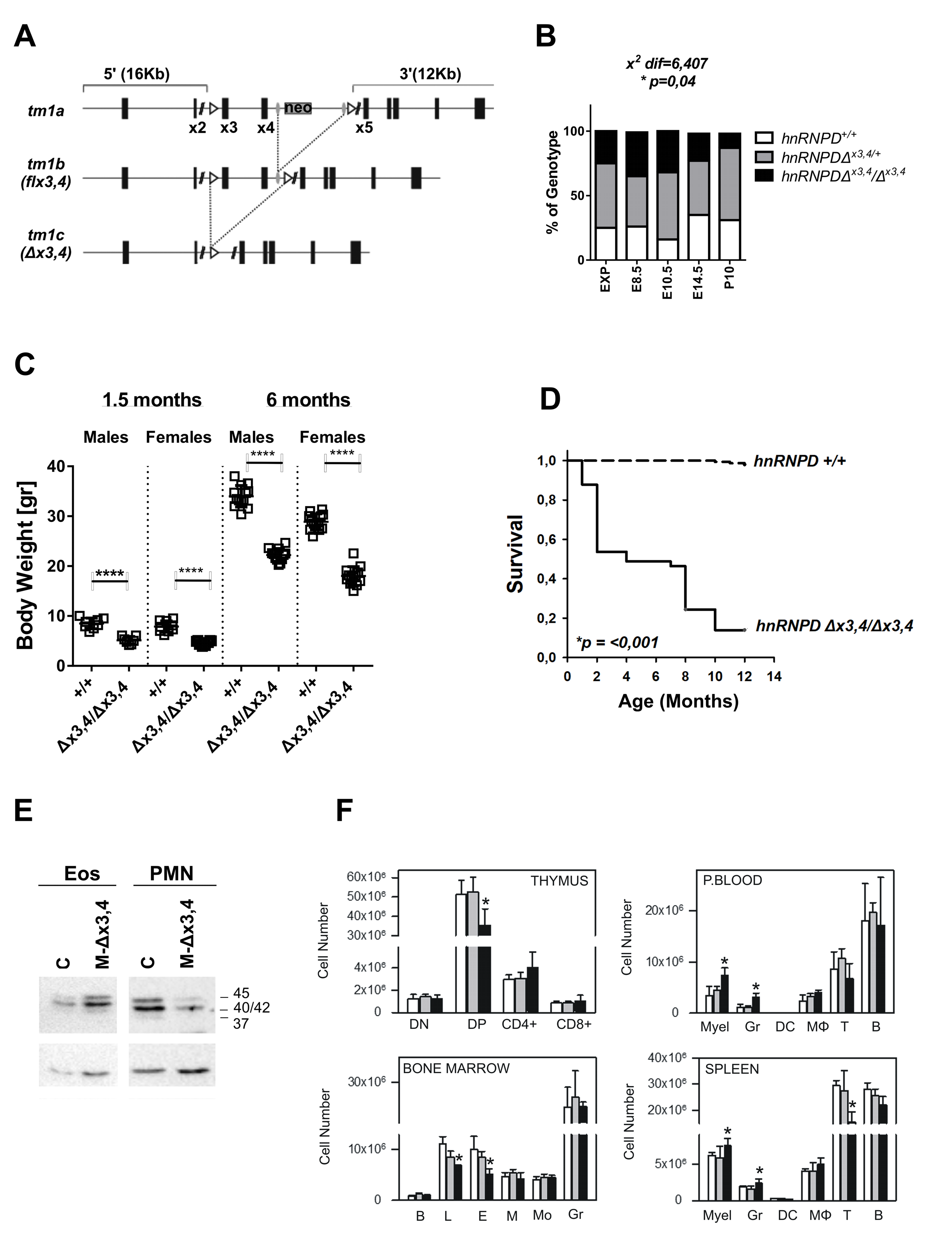


**Supplementary Figure S1.** **Generation and analysis of whole body and myeloid *hnRNPD Δx3,4* mutant mice. (A)** Diagrammatic representation of the targeted *hnRNPD* locus on the mouse chromosome 5 (*tm1a*) in ES cells. Black boxes indicate exons; White triangles represent loxP sites; Grey circles denote FRT recombination sites; and the “neo” box indicates the neomycin acetyltransferase gene used for the antibiotic selection of targeted ES cells. The final allele with the loxP-flanked exons 3-4 (*tm1b*) was generated following the FLPe mediated removal of the FRT-flanked “neo” gene. The expression of Cre recombinase -in either the germline or in myeloid progenitors- leads to the removal of the loxP-flanked exons 3-4 (allele *tm1c*). **(B)** Bar graphs demonstrating the frequency of *hnRNPD^+/+^*, *hnRNPD^Δx3,4/+^* and *hnRNPD^Δx3,4/Δx3,4^* genotypes in embryos from *hnRNPD^Δx3,4/+^* matings at embryonic days E8.5, E10 & E14.5 and pups at postnatal day 10 (P10) and relative to the expected mendelian segregation of alleles (EXP). Data derived from genotyping screens of progeny from >31 pregnancies. *χ2* test statistics for the frequency of *hnRNPD^Δx3,4/Δx3,4^* genotype at P10 are indicated. **(C)** Scatter plots indicating individual body weights of male and female, *hnRNPD^+/+^* and homozygous *hnRNPD^Δx3,4/Δx3,4^* mice and at 1.5 and 6 months of age. (****) denote p<0.0001. **(D)** Kaplan-Meyer distribution and statistics depicting differences in the cumulative survival of *hnRNPD^Δx3,4/Δx3,4^* mice over a period of 14 months (*hnRNPD^+/^*^+^; n=152, *hnRNPD^Δx3,4/Δx3,4^*; n=41). **(E)** Representative immunoblots for the detection of AUF1 proteins in extracts from sorted CD11b^+^SiglecF^+^Gr1^-^ eosinophils and CD11b^+^SiglecF^-^Gr1^+^ polymorphonuclear cells harvested from the peritoneal cavities of *hnRNPD^flx3,4/flx3,4^* controls and *LysMCre+hnRNPD^flx3,4/flx3,4^* *(M-Δx3,4)* mice at 48hrs post the intraperitoneal administration of thioglycolate broth. GAPDH is shown as a loading control. **(F)** Absolute enumeration of central immune subsets in the bone marrow, thymus, peripheral blood and spleen of *hnRNPD^+/+^* and *hnRNPD^flx3,4/flx3,4^* control mice (combined, white bars); *LysMCre+hnRNPD^flx3,4/flx3,4^* mice (gray bars); and *hnRNPD^Δx3,4/Δx3,4^* mice (black bars). Bar graphs depict mean values (+SD) from n=3-6 mice per group at the age of 8 weeks. (*) denote p<0.05 assessed via unpaired Students-t test. Shown are: double negative (DN), double positive (DP), and single positive cells in the thymi following staining with aCD4 and aCD8 antibodies; blast cells (B), lymphoid cells (L), erythroid cells (E), Monocytes (Mo), Granulocytes (Gr) and other myeloids (M) in bone marrows following staining with aCD34 and aLy6C antibodies; total myeloid cells, granulocytes, dendritic cells, macrophages, T and B lymphocytes in the spleens and peripheral blood following staining with aCD11b, aCD11c, Gr1, F480, aCD4 & aCD8 antibodies.


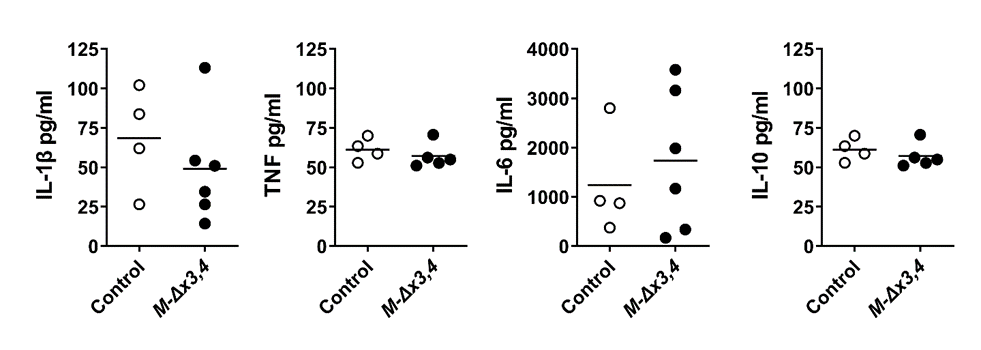


**Supplementary Figure S2.** Detection of cytokines in supernatants from 24hr cultures of colon explants derived from control and M-Δx3,4 mice on day 13 of the DSS protocol. Scatter plots indicate individual and mean protein values (line) as detected via cytokine specific ELISAs.


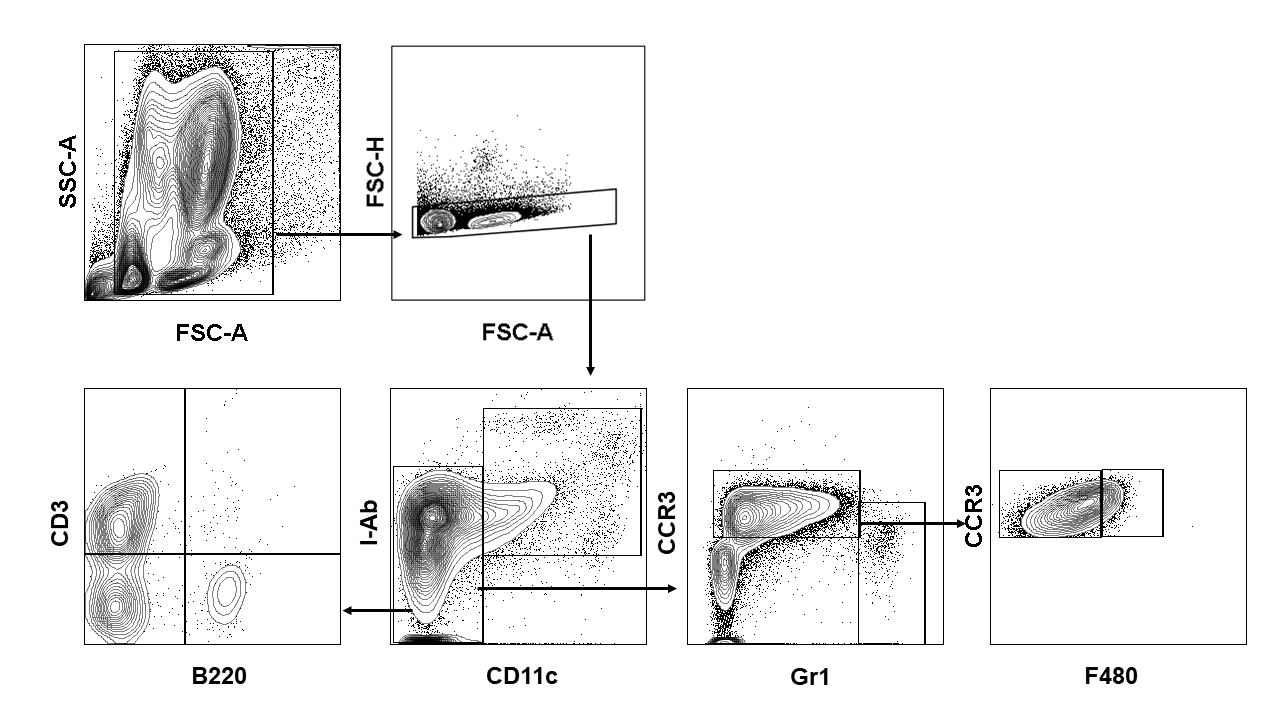


**Supplementary Figure S3.** Gating strategy in PI positive cells for the identification of eosinophils (MHCII^+^CD11c^-^GR1^lo^CCR3^hi^F4/80^lo^), neutrophils (MHCII^+^CD11c^-^CCR3^ind-lo^GR1^hi^), macrophages (MHCII^+^CD11c^-^F4/80^hi^), T cells (MHC-II^lo^CD3^+^B220^-^), and B cells (MHC-II^lo^CD3^-^B220^+^) in single cells from the BALF of mice challenged for OVA-induced allergic airway inflammation as presented in **Figure 3E**.


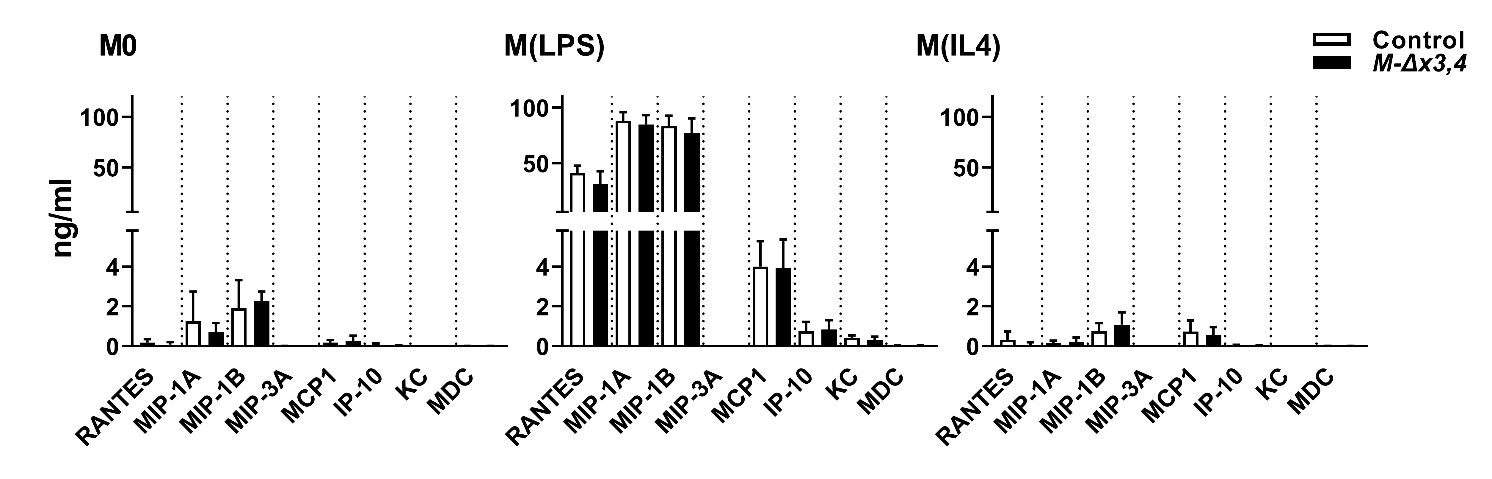


**Supplementary Figure S4.** Detection of secreted RANTES, MIP-1A, MIP-1B, MIP-3A, MCP-1, IP-10, KC and MDC in culture supernatants from Control and M-hnRNPDΔx3,4 BMDMs stimulated with LPS or IL4 for 24hrs and relative to resting (M0). Bar graphs depict mean values (±SD) of protein levels as assessed by cytometric bead arrays.


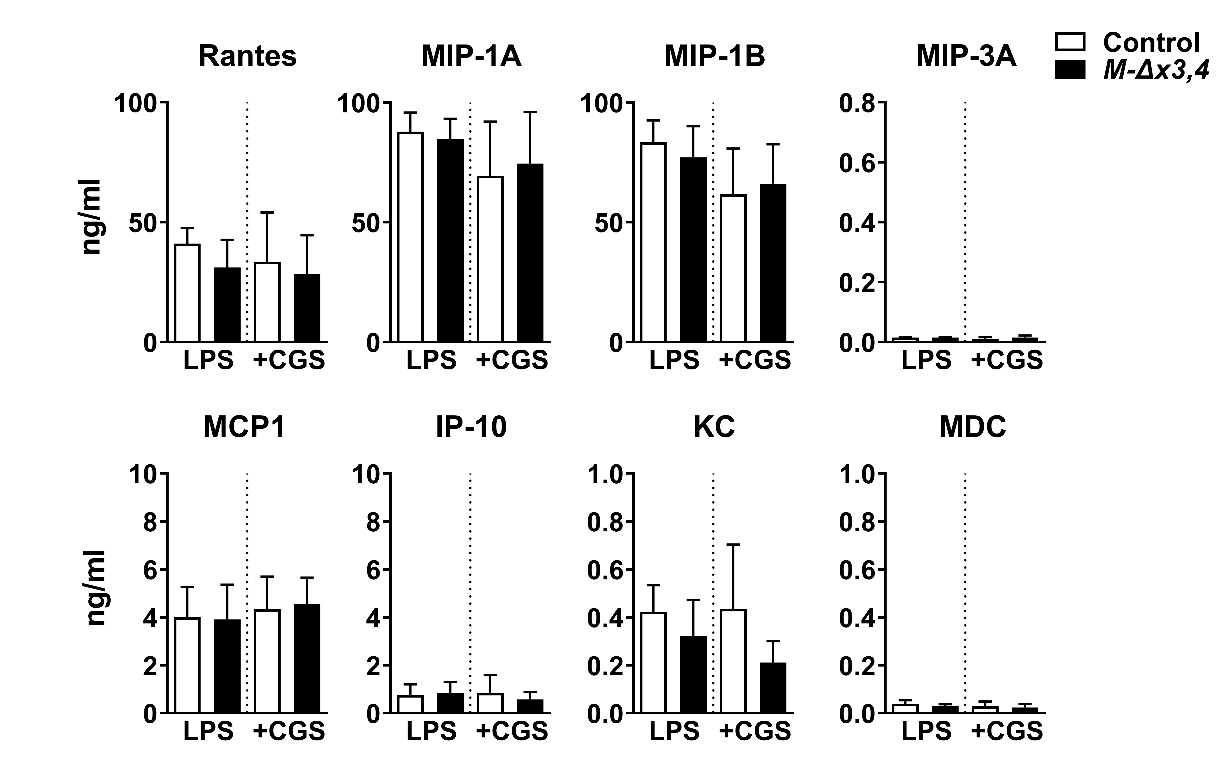


**Supplementary Figure S5**. Detection of Detection of secreted RANTES, MIP-1A, MIP-1B, MIP-3A, MCP-1, IP-10, KC and MDC in culture supernatants from Control and *M-hnRNPDΔx3,4* BMDMs stimulated with LPS or LPS+CGS for 24hrs and relative to resting (M0). Bar graphs depict mean values (±SD) of protein levels as assessed by cytometric bead arrays.


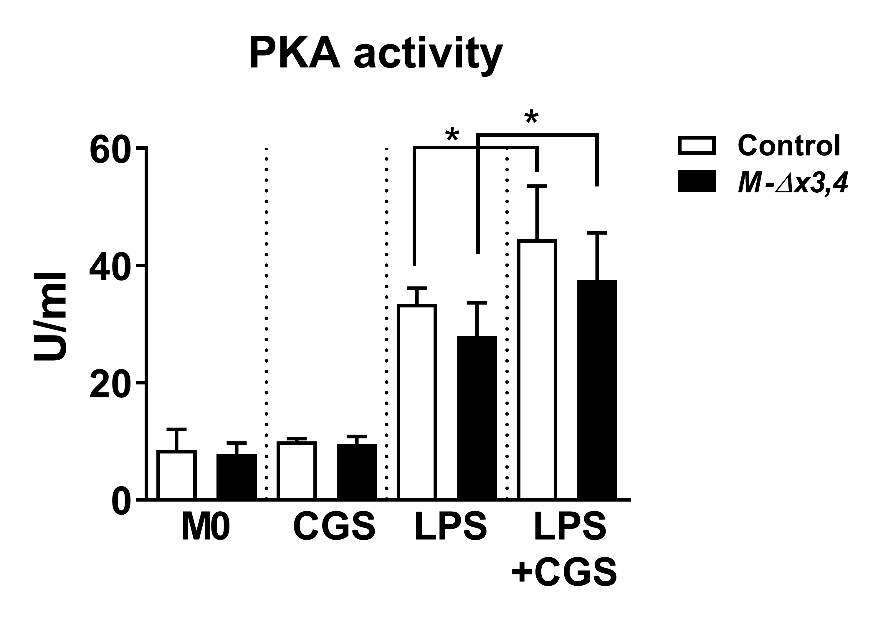


**Supplementary Figure S6**. Detection of Protein Kinase A (PKA) activity in cell lysates of Control and M-hnRNPDΔx3,4 BMDMs stimulated with CGS, LPS or LPS+CGS for 30 min and relative to resting (M0). Bar graphs depict mean values (±SD) of PKA activity as assessed by a colorimetric activity assay.

**
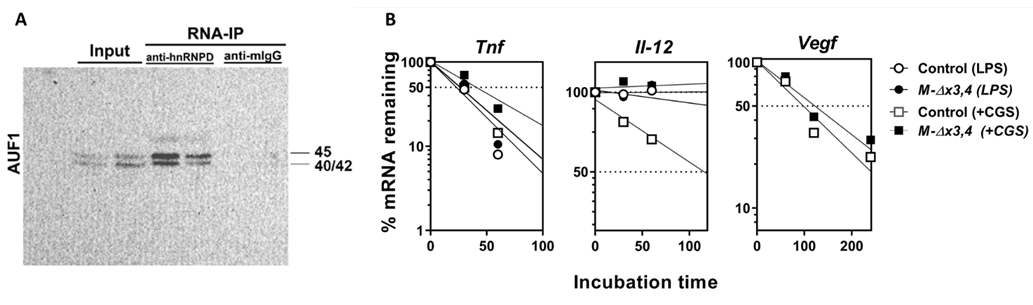
**

**Supplementary Figure S7. RNA-immunoprecipitations and mRNA-decay studies (A**) Representative immunoblot for the detection of AUF1 proteins in the AUF1-RIP material the corresponding extracts relative to control IgG pull-downs. Such material was used to assess interactions of the mRNA transcripts in **Figure 7A.** For western blotting a polyclonal anti-AUF1 antibody (Millipore 07-260) and a IP conformation-specific secondary antibody (anti-mouse IgG -HRP-conjugated Veriblot for IP Detection Reagent, Abcam, ab131366) were used. **(B)** Semilogarithmic plots of normalized qRT-PCR mean data for the *Tnf*, *il12b* and *Vegf* mRNAs in actinomycin D treated macrophages. The half-lives for individual biological replicas are presented in **Figure 7E.**

## 2. Supplementary Methods and Tables

**Cytometric bead arrays for chemokines.**

Expression profiling of the secreted pro-inflammatory chemokines in the culture of activated macrophages (BMDMS) was performed using a cytometric bead-based assay panel following manufacturer’s instructions (Biolegend, Legendplex Panel, Cat. No. 740451). Totally 8 chemokines, including RANTES (CCL5), MIP-1A (CCL3), MIP-1B (CCL4), MIP-3A (CCL20), IP-10 (CXCL10), MCP-1 (CCL2), KC (CXCL1) and MDC (CCL22) were quantitated in the culture supernatants of Control and *M-hnRNPD^Δx3,4^* BMDMs in the resting state (Μ0) or 24 hours post stimulation with LPS (10ng/ml), IL4 (10ng/ml) or LPS (10ng/ml) +CGS (10μΜ). Results are presented in **Figure S3**.

**Library preparation and alignments.**

Library preparation was performed using the QuantSeq 3’ mRNA-Seq Library Prep Kit FWD (QuantSeq-LEXOGEN™, Vienna, Austria), according to manufacturer’s instructions. Briefly, up to 500ng from each RNA sample were used for first strand synthesis. The RNA was subsequently removed and 2nd strand synthesis was initiated by a random primer, containing Ion Torrent compatible linker sequences and appropriate in-line barcodes. 2nd strand synthesis was followed by magnetic bead-based purification and the resulting library was PCR-amplified for 14 cycles and re-purified. Library quality and quantity was assessed on a Bioanalyzer using the DNA High Sensitivity Kit reagents and protocol (Agilent Technologies). The quantified libraries were pooled together at a final concentration of 100pM. The pools were templated and enriched on an Ion Proton One Touch system. Templating was performed using the Ion PI™ Hi-Q™ OT2 200 Kit (Thermo Fisher Scientific), followed by sequencing using the Ion PI™ Hi-Q™ Sequencing 200 Kit on Ion Proton PI™ V2 chips (Thermo Fisher Scientific), an Ion Proton™ System, according to the manufacturer's instructions. The quality of Quant-Seq FASTQ files obtained from Ion Proton sequencing were assessed using FastQC. Reads were mapped on the UCSC mm10 reference genome using a two-phase mapping procedure: short reads were mapped using TopHat2 v.2.1.1 (1) and the reads which remained unmapped were submitted to a second round of mapping using Bowtie2 (2) v.2.3.3 against the mm10 genome with the ‘—local’ and ‘--very-sensitive-local’ switches turned on, after re-conversion of BAM to FASTQ files using BEDTools (3) suite v.2.28.0 bam2fastq function. Sorting and indexing of the merged reads was performed using SAMtools (4) v.1.9. All resulting BAM files were visualized in the UCSC Genome Browser using BEDtools and tools provided by the UCSC Genome Browser toolkit. The resulting Quant-Seq BAM files were analyzed on a transcript level base with the Bioconductor package metaseqR2(79) v.1.3.14 which has built-in support for Quant-Seq data.

**Bioinformatics analysis**

Mapped sequences (Quant-Seq BAM files) were analyzed on a transcript level base with the Bioconductor package metaseqR2(76) v.1.3.14 which has built-in support for Quant-Seq data. Briefly, the raw BAM files, one for each sample, were summarized to a 3’ UTR read counts table from Ensembl longest (dominant) transcripts (version 90). The original 3’ UTR regions were extended 500bp upstream and downstream to accommodate the variable read length of Ion Proton reads. In the resulting read counts table, each row represented one 3’ UTR region, each column one Quant-Seq sample and each cell the corresponding read counts associated with each row and column. The final 3’ UTR read counts table was normalized for inherent systematic or experimental biases using the Bioconductor package DESeq2 after removing areas that had zero counts over all the Quant-Seq samples. Prior to the statistical testing procedure, the 3’ UTR read counts were filtered for possible artifacts that could affect the subsequent statistical testing procedures. 3’ UTR areas presenting any of the following were excluded from further analysis: i) 3’ UTR areas corresponding to genes less than 500bp, ii) 3’ UTRs with read counts below the median read counts of the total normalized count distribution. Similar expression thresholds (e.g. the median of the count distribution) have been previously used in the literature, where the authors use the median RPKM value instead of normalized counts), iii) 3’ UTR areas corresponding to genes with the following Ensembl biotypes: rRNA, TR_V_pseudogene, TR_J_pseudogene, IG_C_pseudogene, IG_J_pseudogene, IG_V_pseudogene. The remaining 3’ UTR counts table after filter application was subjected to differential expression analysis for the contrasts MΔχ3,4 versus Control utilizing DESeq2, edgeR, NOISeq, limma, NBPSeq, ABSSeq and DSS algorithms and a combined meta-analysis procedure by the PANDORA algorithm implemented in metaseqR2. 3’ UTR areas (and their corresponding transcripts) presenting a PANDORA p-value less than 0.05 and an absolute fold change (for each contrast) greater than 1 in log2 scale were considered as differentially expressed.

**Colon explant cultures.**

Colon explant cultures were performed as previously described (5). Briefly, whole colons were isolated and washed with PBS supplemented with 20 mg/ml gentamycin to remove residual intestinal bacteria and cut in 1.5 cm pieces into a 48-well plate containing 500 μl RPMI-1640 per well. Tissues were incubated at 37°C, 5% CO2 for 24 h, and supernatants were collected for cytokine ELISA measurements.

**PKA activity**

PKA (Protein Kinase A) activity was measured in cell lysates from Control and M-hnRNPDΔx3,4 BMDMs stimulated with CGS, LPS or LPS+CGS for 30 min using PKA colorimetric activity Kit (Invitrogen™ EIAPKA). Procedure was performed following manufacturer's instructions.

## List of primers used for qRT-PCR.

| **Primers for RT-PCR** | **Sequence5’->3’** |
| --- | --- |
| *mHnRNPD exon 3 S* | ATGTTTATAGGAGGCCTTAG |
| *mHnRNPD exon  3 A* | AGCACAAAGCCAAAACCCCT |
| *mHnRNPD exon 6 S* | GTAGCCATGTCAAAGGAACA |
| *mHnRNPD exon 6 A* | ACCCTCCTCTAGATCCCAC |
| *mHnRNPD exon 4-6 S* | GTAGCCATGTCAAAGGAACA |
| *mHnRNPD exon 4-6 A* | GTGCCATGTCAAAGGAACA |
| *mB2M S* | TTCTGGTGCTTGTCTCACTGA |
| *mB2M A* | CAGTATGTTCGGCTTCCCATTC |
| *mGadph S* | TGCACCACCAACTGCTTAGC |
| *mGadph A* | GGCATGGACTGTGGTCATGAG |
| *mTnf S* | CACGCTCTTCTGTCTACTGA |
| *mTnf A* | ATCTGAGTGTGAGGGTCTGG |
| *mIl12b S* | TGTCCTCAGAAGCTAACCAT |
| *mIl12b A* | CCAGTCCACCTCTACAACAT |
| *mIl6 S* | CTTCTTGGGACTGATGCTGGTGAC |
| *mIl6 A* | TCCAGGTAGCTATGGTACTCCAGA |
| *mArg1 S* | CCAGAAGAATGGAAGAGTCAGTGT |
| *mArg1 A* | GCAGATATGCAGGGAGTCACC |
| *mRetnla/Fizz1 S* | CTGCCCTGCTGGGATGACT |
| *mRetnla/Fizz1 A* | CATCATATCAAAGCTGGGTTCTCC |
| *Chi3l3/Ym1 S* | CAAGTTGAAGGCTCAGTGGCTC |
| *Chi3l3/Ym1 A* | CAAATCATTGTGTAAAGCTCCTCTC |
| *mMrc1 S* | ACTGCGTGGTTATGAAAGGC |
| *mMrc1 A* | TGAGCGACGAGTACAAGATGC |
| *mVegf S* | TTACTGCTGTACCTCCACC |
| *mVegf A* | ACAGGACGGCTTGAAGATG |
| *mThbs1 S* | ACTTCACCTTTGCCACCTC |
| *mThbs1 A* | AGACTCTGGAATGCGGTT |
| *mCcl2 S* | AGCACCAGCACCAGCCAACT |
| *mCcl2 A* | TTCCTTCTTGGGGTCAGCAC |
| mAdora2a A | GCCATCCCATTCGCCATCA |
| mAdora2a S | GCAATAGCCAAGAGGCTGAAGA |
| mAdora2b A | GCGTCCCGCTCAGGTATAAAG |
| mAdora2b S | CCCCAGGAACGGAGTCAATC |

## Supplementary references.

1. Kim D, Pertea G, Trapnell C, Pimentel H, Kelley R, Salzberg SL. TopHat2: accurate alignment of transcriptomes in the presence of insertions, deletions and gene fusions. Genome Biol. 2013;14(4):R36.

2. Langmead B, Salzberg SL. Fast gapped-read alignment with Bowtie 2. Nat Methods. 2012;9(4):357-9.

3. Quinlan AR, Hall IM. BEDTools: a flexible suite of utilities for comparing genomic features. Bioinformatics. 2010;26(6):841-2.

4. Li H, Handsaker B, Wysoker A, Fennell T, Ruan J, Homer N, et al. The Sequence Alignment/Map format and SAMtools. Bioinformatics. 2009;25(16):2078-9.

5. Christodoulou-Vafeiadou E, Ioakeimidis F, Andreadou M, Giagkas G, Stamatakis G, Reczko M, et al. Divergent Innate and Epithelial Functions of the RNA-Binding Protein HuR in Intestinal Inflammation. Front Immunol. 2018;9:2732.
